# Drought- and heat-induced mortality of conifer trees is explained by leaf and growth legacies

**DOI:** 10.1126/sciadv.adl4800

**Published:** 2024-04-12

**Authors:** Frank J. Sterck, Yanjun Song, Lourens Poorter

**Affiliations:** ^1^ Forest Ecology and Forest Management Group, Wageningen University and Research, P.O. Box 47, 6700 AA Wageningen, Netherlands.; ^2^ School of Biological Sciences, Washington State University, P.O. Box 644236, Pullman, WA 99164-4236, USA.

## Abstract

An increased frequency and severity of droughts and heat waves have resulted in increased tree mortality and forest dieback across the world, but underlying mechanisms are poorly understood. We used a common garden experiment with 20 conifer tree species to quantify mortality after three consecutive hot, dry summers and tested whether mortality could be explained by putative underlying mechanisms, such as stem hydraulics and legacies affected by leaf life span and stem growth responses to previous droughts. Mortality varied from 0 to 79% across species and was not affected by hydraulic traits. Mortality increased with species’ leaf life span probably because leaf damage caused crown dieback and contributed to carbon depletion and bark beetle damage. Mortality also increased with lower growth resilience, which may exacerbate the contribution of carbon depletion and bark beetle sensitivity to tree mortality. Our study highlights how ecological legacies at different time scales can explain tree mortality in response to hot, dry periods and climate change.

## Teaser

Leaf and growth legacies explain dry and hot summer-induced mortality differences across conifer species.

## INTRODUCTION

**Fig. 1.**
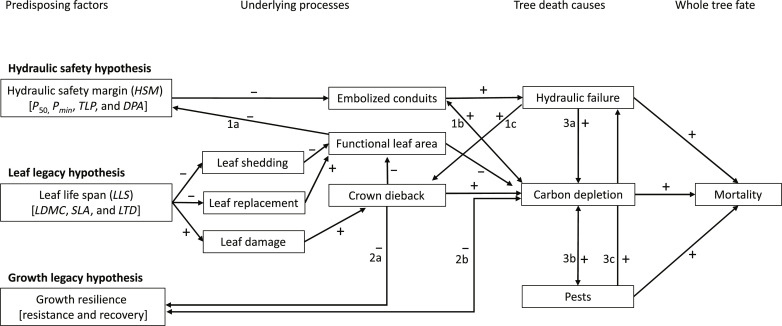
A conceptual framework for explaining tree mortality. The framework shows how legacy effects of hydraulic safety margin (*HSM*; short term, days to weeks), leaf life span (*LLS*; intermediate term, months to years), and growth resilience (years to decades) may contribute to drought- and heat-induced tree mortality through underlying processes and their implications for the main mechanisms causing tree death. Arrows refer to expected negative (−) or positive (+) effects. Numbers refer to feedback loops between the hydraulic safety and leaf legacy hypotheses (1a to 1c), leaf legacy and growth legacy hypotheses (2a and 2b), and interdependencies among three main potential causes of tree death (3a to 3c). The predisposing factors are probably associated with other traits, with abbreviations mentioned between parentheses. Hydraulic traits include embolism resistance *P*_50_, which is the branch water potential causing 50% loss in water conductance after the spread of air bubbles (emboli); pit diameter aperture (*DPA*) (*32*); the minimum leaf water potential observed during a severe, dry period (*P*_*min*_); the turgor loss point (*TLP*; the water potential at which leaves lose their turgor). The *HSM* was calculated as *P*_*min*_ − *P*_50_. Leaf traits associated with *LLS* include leaf dry matter content (*LDMC*; the leaf dry mass divided by the leaf fresh mass), specific leaf area (*SLA*; leaf area per leaf mass), and leaf tissue density (*LTD*; the leaf dry mass per unit fresh leaf volume). The growth resilience is determined by the growth resistance (stem growth reduction during a drought year) and growth recovery after the drought year (see Material and Methods for measurements and calculations).

Across the globe, climate change has led to an increased frequency and severity of drought and heat waves (*1*) and increased plant drought and heat stress. This has resulted in a reduced crown-vitality (*2*), growth and survival of trees (*3*), and increased forest dieback over the past decades (*4*, *5*). Such forest dieback may impair ecosystem functioning and wood production, result in biodiversity loss, and turn forests from a carbon sink into a carbon source (*6*). The future of many forests is therefore at stake (*7*), yet, our ability to predict the forest’s future is limited since we poorly understand the factors that underlie drought- and heat wave–induced tree mortality (*8*, *9*). One reason for this is that tree mortality is a complex process since it involves mechanisms that operate at different scales in space [from cells to roots, stem, and leaves to whole plant (*9*)] and time [from short-term hydraulic failure to longer-term crown dieback followed by tree death (*10*)] and it is difficult to integrate them (*11*). Here, we evaluate three hypotheses for drought- and heat wave–induced tree mortality highlighting the implications of species differences in hydraulic traits, leaf traits, and growth resilience (Fig. 1). We test these hypotheses using a long-term comparative study where tree mortality, hydraulic traits, leaf traits, and stem growth responses to drought have been quantified for 20 coniferous tree species.

Drought-induced tree mortality has been attributed to three different, interacting, mechanisms (Fig. 1) (*12*): (i) hydraulic failure which occurs when conduits embolize (become air-filled) because hydraulic safety margins (*HSM*) cannot be maintained and lead to lethal effects of dehydration, including irreversibly damaged cells and meristem death leading to crown dieback and mortality (*13*); (ii) depletion of carbon reserve pools reducing metabolic functioning and investments in maintenance, defense (*14*), and/or embolism repair (*15*); and (iii) pests that may kill trees weakened by carbon depletion or hydraulic failure (*12*). Many factors potentially predispose such causes of tree death in complex, interactive ways and are poorly understood (*11*). Here, we present three non-mutually exclusive hypotheses about the possible roles of such predisposing factors, which represent physiological legacies potentially lasting from days, years, to decades.

First, the hydraulic safety hypothesis postulates that failure of the water transporting system leads to plant death. Droughts may lead to continued water loss via leaky leaf stomata, leaf cuticles, or stem bark (*16*) after stomata close (*17*), causing increasingly (negative) water potentials in the water-transporting wood. Beyond a critical threshold, the tension resulting from negative water potentials may pull air bubbles (embolisms) into water transporting conduits, impair water transport, and lead to the desiccation of branches and leaves, causing crown dieback and eventually tree mortality (*10*, *18*). Trees may avoid such hydraulic failure by maintaining wide *HSM*s, with *HSM* quantified as the difference between the lowest water potential (*P*_*min*_) experienced by branches or leaves and the water potential at which tree branches lose 50% hydraulic conductivity (*P*_50_). A recent meta-analysis indicates that hydraulic failure may often precede drought-induced mortality (*19*), although the predictive role of *HSM* or related hydraulic traits for tree mortality varies across forests [see, e.g., (*20*–*22*)].

Second, the leaf legacy hypothesis postulates that species with short-lived leaves are better able to replace drought- or heat-damaged and shed leaves, and track changes in environmental conditions (Fig. 1) (*23*). Severe droughts may cause dehydration and, in combination with heat waves, irreversible leaf tissue damage (Fig. 1) (*24*). Alternatively, trees may actively shed leaves, thus reducing water loss and embolism risks, and maintaining a hydrated and functional crown (*8*). Both leaf damage and shedding come at the cost of a loss of leaf biomass, nutrients, and photosynthetic leaf area and, hence, lead to a reduction in current and/or future carbon gain (*25*). Species with cheap short-lived leaves may rapidly replace their leaf population and track annual dynamics in environmental conditions (*23*). In contrast, species with long-lived leaves, which are usually more expensive, may less easily replace damaged or lost leaves because their crown contains multiple, accumulated leaf cohorts; each year only a relatively small number of leaves is produced and canopy turnover is therefore slow. As a result, they may recover more slowly in leaf area and growth (*26*). We hypothesize that tree species with longer leaf life span (*LLS*) face higher mortality risks in response to hot, dry periods due to legacies of leaf damage or leaf loss by crown dieback. Yet, the potential implications of *LLS* in explaining tree mortality risks remain untested (*27*).

Third, the growth-legacy hypothesis postulates that previous growth resilience responses to drought express tree vigor and the ability of trees to survive future droughts (Fig. 1) (*27*). Trees are long-lived organisms that face multiple droughts during their life. In general, trees reduce growth during extreme dry years but rapidly recover growth after such dry years (*28*). Conifer trees may, however, show slow growth recovery and in turn low resilience, and those trees typically face higher mortality risks during future droughts than conspecific conifer trees with rapid growth recovery (*27*). Growth recovery may be particularly critical for conifers because they have less storage parenchyma and therefore relatively low levels of carbohydrate reserves in the wood compared to angiosperm trees (*29*). Conifers may therefore face a limited capacity to pay for the growth of new wood, thus limiting growth recovery after drought. The implications of growth resilience differences in explaining mortality risks across different tree species remain so far untested.

We thus hypothesized that tree species differ in their mortality responses to drought and heat waves because of differences in hydraulic traits, *LLS*, and/or growth resilience. We predict that mortality risks will be higher for tree species with (i) narrower *HSM*s and/or hydraulic traits indicating higher risks for embolism, (ii) higher *LLS* and associated leaf traits indicating high costs for leaf area recovery and reduced ability to track environmental dynamics, and (iii) lower growth resilience to droughts before the lethal drought. We use a conceptual framework (Fig. 1) to discuss the possible mechanisms driving the drought- and heat-induced tree mortality for each of the predictions separately and, after that, discuss the possible feedback loops and synergies between these different mechanisms of drought- and/or heat-induced tree mortality.

We test our predictions for 20 conifer tree species that are grown in monospecific stands in a common garden experiment in Netherlands since at least 1970 (table S1). Such a common garden approach excludes confounding geographic effects of macroclimate and soils on species’ responses to drought. In the winter covering the transition between 2017 and 2018, we sampled the stem cores to quantify stem growth resilience responses using ring data, and in the summer of 2018, we measured the *HSM*s and related hydraulic traits, *LLS*, and associated leaf traits. The stem growth resilience responses include the growth reduction during dry years and recovery during follow-up years and were measured from stem growth responses to the dry summers between 1970 and 2018 (figs. S1 and S2) (*26*). To assess tree mortality, we took advantage of a natural experiment, in which the hot and dry summer of 2018 caused negative impacts on the growth and survival of trees across Central Europe (*30*). Tree mortality in the common garden experiment was probably triggered by the year 2018 (fig. S2) and possibly enforced by the summers of 2019 and 2020, which were also drier than average summers (*31*). The conifer species were growing in monospecific stands. To quantify tree mortality after these hot and/or dry summers, we counted in spring 2021 for each monospecific stand the number of dead trees relative to all dead and alive trees present. Since we did not have an a priori idea of this sequence of droughts and its implication for tree mortality, we had not monitored crown dynamics or carbon depletion from 2018 to 2021 but speculated on their roles following our conceptual framework. Our study shows that tree mortality differences across conifer tree species following hot and dry summers were driven by species-specific leaf and growth legacies, and not by species-specific hydraulic traits.

## RESULTS

### Mortality risk was not related to hydraulic traits

**Fig. 2.**
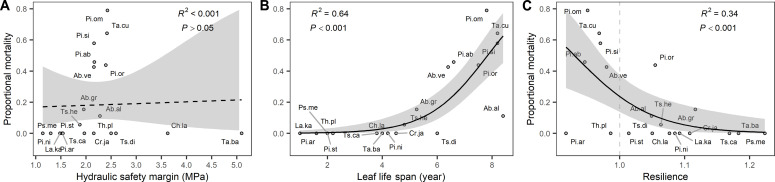
Conifer tree mortality patterns. Conifer species comparisons for patterns of mortality in response to the 2018 drought year with (**A**) the *HSM*, (**B**) *LLS*, and (**C**) growth resilience (*N* = 20 species). For each of the three hypotheses (see Fig. 1), we show the trait that best explains the predicted mechanism driving tree mortality. For the other associated traits, see figs. S3 to S5. Species abbreviations are explained in table S1. The shown result for resilience included six droughts preceding the 2018 drought year (from 1986 onward): The result for a longer time series of droughts (including 1975–1976 and 1982–1983) is presented in fig. S8 and was used in (*26*, *28*) but did not include *Abies veitchii* and *P. armandii* for one or two of these early droughts.

Tree mortality following the hot and dry 2018 summer varied from 0 to 79% across species and was not significantly related to *HSM* (Fig. 2A) and its underlying components, the twig embolism resistance and minimum twig water potential (fig. S3, B and C). Mortality was neither related to other hydraulic traits such as pit aperture diameter—a key driver of embolism resistance in these species (*32*)—nor to turgor loss point (*TLP*; fig. S3). Trees maintained leaf water potentials at relatively high levels (species average, −1.84) within a relatively narrow range (−2.42 to −1.42 MPa) and their positive *HSM* values (species average, 2.24; range, 1.14 to 5.10 MPa) indicate that hydraulic failure was avoided during the extremely dry and hot summer of 2018.

### High mortality risks for species with longer leaf life spans

Mortality risks increased nonlinearly with *LLS* and were especially higher for species with maximum *LLS* >5 years (*r*^2^ = 0.64; Fig. 2B), although *Abies alba* had a relatively low mortality (11%) compared to other species with such long *LLS*s. Other leaf traits showed no significant relationships with tree mortality (fig. S4 and table S2). *LLS* was positively correlated with leaf tissue density and tended to be negatively correlated with specific leaf area (*SLA*) (Pearson *r* = −0.42, *P* = 0.06, double-sided; table S2). Species coming from colder areas had longer *LLS*s as *LLS* was negatively correlated with mean annual temperature (Spearman’s correlation, *r* = −0.46) and the temperature of the warmest month (*r* = −0.64) in the area of origin (Table 1). Species coming from colder areas (*MAT*) with lower transpiration demands (*PET*) also showed higher mortality (Table 1).

**Table 1.** Correlations between climate niches with mortality and traits across species. Spearman’s correlation coefficients associating the climate niches with mortality risks and functional traits across 20 conifer species studied in a common garden experiment in Netherlands. MAT and *T*_max_ stand for the mean annual temperature and the maximum monthly temperature in the geographic distribution range of each species respectively; *MAP* and *PR*_*min*_ for the mean annual precipitation and minimum monthly rainfall respectively; and *PET* and *PET*_*max*_ for the mean potential evapotranspiration and maximum monthly potential evapotranspiration. Significant correlations [*N* = 20 (tree species), *P* < 0.05] are in bold. The climate distribution ranges of the species were obtained from the Global Biodiversity Information Facility GBIF: https://gbif.org/ [for details, see (*28*)].

Climate limits	Mortality	HSM	Leaf life span	Growth resilience
* MAT*	**−0.45**	−0.34	**−0.46**	0.28
* T *_*max*_	**−0.65**	−0.39	**−0.64**	0.31
* MAP*	−0.14	0.12	−0.01	−0.08
* PR*_* min*_	−0.10	0.21	0.13	−0.07
* PET*	**−0.60**	**−0.56**	**−0.63**	**0.53**
* PET*_* max*_	−0.37	−0.24	−0.35	**0.50**

### Mortality risks increased with lower growth resilience

Our results show that mortality following the hot and dry summers decreased nonlinearly with the growth resilience to drought averaged for the 6 dry years from 1986 to 2013 (*r*^2^ = 0.34; Fig. 2C and figs. S1 and S2). The mortality responses were more strongly related to growth recovery than growth resistance (fig. S5). In particular, species with incomplete growth resilience to previous droughts (i.e., <1) faced higher mortality risks (>30%; Fig. 2C).

### Leaf and growth legacy effects enforce each other

A multiple regression model including effects of *HSM*,* LLS*, and growth resilience explained 84% of the mortality differences across species (Table 2), also when accounting for possible collinearity between growth resilience versus *LLS* (table S3). These results show that the effects of leaf and growth legacies were significant, additive, and enforced each other, while *HSM* did not have a significant effect.

**Table 2.** Explaining mortality from possible underlying traits. Test statistics (regression coefficients and probability values) of a multiple logistic regression model predicting the mortality risks from the *HSM*, *LLS*, and growth resilience (*Resilience*) across 20 conifer tree species. For visualizations of the logistic regression models per individual trait, see Fig. 2. When bark beetle presence (table S1) and alternatively *Picea* (as this genus seems highly sensitive to bark beetle attack in general) were added as factors, they did not significantly contribute to the observed variation and were therefore excluded from the shown analysis. Yet, bark beetle presence was associated with *LLS* and growth resilience, but not *HSM*s (fig. S9). Residuals did not deviate from normality (Shapiro’s *W* = 0.946, *P* = 0.31, *N* = 20). An alternative model was carried out to account for possible collinearity between growth resilience and *LLS* (table S3): It gave similar results, confirming the additive contributions of *LLS* and growth resilience.

	Independent variables			Intercept	*R^2^*
	* HSM*	* Leaf life span*	* Resilience*		
Coefficients	−0.34	1.81	−1.24	−3.02	0.84
*P* values	0.70	<0.001	0.01	<0.001	<0.001

## DISCUSSION

### Mortality risk was not related to hydraulic traits

This is a rather surprising result, as the main proxies of hydraulic failure, especially *HSM* and embolism resistance, are generally thought to contribute to species mortality in response to drought (*19*, *20*). Even during the severest dry period in 2018, trees maintained relatively high leaf water potentials (species average, −1.84; range, −2.42 to −1.42 MPa) and their *HSM*s were therefore sufficiently positive and wide (species average, 2.24; range, 1.14 to 5.10 MPa) to avoid hydraulic failure during that extremely dry and hot summer. These *HSM* values are in line with values reported for gymnosperms worldwide, and larger than those reported for angiosperm trees (usually <1 MPa) in different biomes (*33*).

Our dataset includes multiple Pinaceae and Cupressaceae species. Pinaceae are known to be relatively sensitive to hydraulic failure (*34*) and the five species with the highest embolism vulnerability in our study (with relatively low *HSM* < 1.5) belonged to the Pinaceae (*Pinus armandii*, *Pinus strobus*, *Pinus nigra*, *Larix kaempferi*, and *Pseudotsuga menziesii*; fig. S6). Yet, these species survived the 2018 drought probably because they could avoid embolisms in shoots (*35*). It is possible that hydraulic failure risks were mitigated by relatively wetter and cooler summer months compared to the original distribution areas of multiple species (fig. S7). Moreover, trees may have root systems adapted to dry soils since the sandy soils are easily drained and the deep soil water table (~10 m) is out of reach for tree roots. While such soil conditions could favor interruptions in the soil root water continuum of trees (*36*) thus cutting off the water supply and risking gradual dehydration (*37*), minimum twig water potentials (measured during the drought/heat wave in 2018) and positive *HSM*s imply that trees avoided lethal embolism levels (*13*) by strong stomatal control during that dry summer. These results confirm that hydraulic traits can be weak predictors for tree mortality (*22*) [but see (*20*)] and imply that alternative traits and mechanisms are required for understanding variation in drought- and heat-induced tree mortality of gymnosperms.

### High mortality risks for species with longer leaf life spans

Mortality risks increased rapidly and nonlinearly with *LLS* but were not significantly related to other leaf traits (fig. S4 and table S2), indicating that especially leaf longevity modulates plant responses to drought. *LLS* showed a negative, near-significant correlation with *SLA*, which confirms that species with cheaper leaves (high *SLA*) tend to be better equipped for replacing leaves rapidly than species with more expensive leaves (low *SLA*). Yet, higher leaf area construction costs (*SLA*) did not explain variation in tree mortality. Following the dry, hot periods, trees may have dropped leaves either triggered by direct damage [e.g., osmotic or photo-oxidative stresses that lead to cellular damage (*38*, *39*)] or as an adaptive response that reduces water loss and contributes to maintaining hydraulic integrity (*8*). Once leaves are damaged and/or lost, this may have stronger repercussions for species with long-lived leaves since they are conservative, grow slowly, and thus have limited ability to replace damaged or lost leaves, but with a minor role for variation in leaf area costs (*SLA*). Tree species with long *LLS* may therefore face crown dieback as observed for multiple species in 2020, reduced carbon reserves, and weakened defense against bark beetle attacks (Fig. 1) (*40*).

In our study, species with longer *LLS*s and high tree mortality rates came from colder areas with lower transpiration demands (Table 1). Species with a long *LLS* may thus be adapted to a cold and short growing season (*41*) and maladapted to relatively high temperatures and high transpiration demands in our study site (cross-species correlations for mortality-*PET* = −0.60, *LLS*-*PET* = −0.63; Table 1). We speculate that such maladaptation intensified leaf damage by, for example, persistent photochemical damage caused by the high temperatures (*42*), as encountered during the 2018 summer (with temperatures >40°C, for drought; see fig. S2), which may have induced leaf shedding and reduced the functional leaf area (Fig. 1). Other studies also show that a combination of being close or beyond natural climate distribution limits and an extreme climate event can contribute to forest die-off (*43*). Our results imply that planting conifer trees outside their original climatic envelope in areas that are—or will be—too warm or dry should therefore be avoided, considering the increased risks of heat waves and drought events with climate change.

### Mortality risks increased with lower growth resilience to previous droughts

Our results show that tree mortality was lower for species with a greater growth resilience to dry years preceding 2018 (Fig. 2C and fig. S8) due to species differences in growth recovery rather than growth resistance (fig. S5). Another study showed that, within conifer species, trees with a lower growth recovery to dry periods faced a higher mortality risk to severe future droughts and that mortality risks were more strongly related to drought recovery than to drought resistance (*27*). Poor growth recovery may result from the depletion of internal carbon reserves under extreme droughts (*44*), slowing down the regrowth of wood (*45*). In contrast, angiosperm trees may suffer less from carbon depletion (*19*) because they have more parenchyma tissue to store carbon but vary more strongly in growth resistance and tend to recover more quickly after extreme dry years (*27*, *44*). Overall, our results indicated that incomplete growth resilience (<1) can act as an early signal for enhanced mortality risks under severe future drought, and our study shows that this works not only within species (*27*) but also across tree species.

### Feedback loops and synergistic effects

The conceptual framework indicates that implications of species differences in hydraulic traits, *LLS*, and associated leaf traits and growth resilience for tree mortality do not operate in isolation but may mutually influence each other via feedback loops (Fig. 1). For example, leaf legacies may influence hydraulics via losses in functional leaf area (Fig. 1, arrow 1a), which was observed but not quantified in some tree populations facing tree mortality. Such leaf shedding, crown dieback, and reduced functional leaf area with lower transpiration may hence lead to a smaller drop in leaf water potential and maintain wide *HSM*s (*17*). Such wide safety margins reduce the risks for embolization of conduits and hydraulic failure, which, in turn, reduce risks for carbon depletion (arrow 1b) and/or crown dieback (arrow 1c). From this perspective, leaf legacy effects may have reduced the role of hydraulic failure in driving mortality differences across the studied conifer species.

Leaf legacies may also influence growth legacies. Such leaf legacies occur when leaf damage leads to crown dieback, which may reduce the growth recovery after drought events because of reduced photosynthetic leaf area (arrow 2a) which curtails photosynthetic capacity and contributes to carbon depletion, as little is being assimilated and stored (arrow 2b). Such feedback loops may explain the negative correlation between *LLS* and growth resilience (table S2) [confirmed by Song *et al*. (*28*)]. Nevertheless, *LLS* and growth resilience acted as additive predictors of tree mortality and the full model explained >80% of the variation in tree mortality (Table 2 and table S3). Overall, these results imply that both leaf and resilience legacies contributed to carbon depletion as a factor explaining the differences in mortality across conifer species.

The three major mechanisms underlying tree death (hydraulic failure, carbon depletion, and pests) also interact, and positive arrows in the framework indicate that they may reinforce each other (Fig. 1) (*12*). For example, hydraulic failure may increase carbon depletion because of impaired water transport and assimilation rates (*12*) (arrow 3a), whereas carbon depletion may exacerbate hydraulic failure, as no energy is available to repair embolized conduits in for example small twigs (*35*) (arrow 1b). Yet, our study implies that such interdependency between hydraulic failure and carbon depletion cannot explain the mortality differences across the conifer species.

Alternatively, synergies may occur between carbon depletion and pest pressure (Fig. 1). On the one hand, carbon depletion may increase pest pressure (arrow 3b), as insufficient energy and carbon are available for defense against pests and pathogens. On the other hand, pest pressure may deplete carbohydrate pools as energy is needed for induced defenses (e.g., resin production) and tissue repair (also arrow 3b) and may also affect the water transport system (arrow 3c). In our study, infestation of multiple species by bark beetles (table S1) indicates that pests—probably in addition to carbon depletion—triggered tree mortality. Since carbon depletion was probably strongest for the species with long-lived leaves and low resilience, these species may be more vulnerable to bark beetles (Fig. 1, arrow 3b). Alternatively, tree species with long *LLS* come from colder areas (Table 1) and thus likely suffer less from bark beetle outbreaks in their natural distribution area. Such tree species from colder areas may be less protected and more vulnerable to bark beetle attacks, which could also contribute to carbon depletion (Fig. 1, arrow 3b). Bark beetles were observed on various species with long *LLS* and low resilience (all four *Picea* species; fig. S9), but not or only incidentally for other species with long *LLS* and high tree mortality (*Taxus cuspidata* and three *Abies* species). Conversely, trees from species with shorter *LLS*s (all species with max. *LLS* (<5 years; Fig. 2B) survived the hot, dry summers, as they apparently maintain resource acquisition and defenses, reducing pest risks. From this, we speculate that both leaf and growth legacies contribute to carbon depletion and pest infestation and, in turn, to tree mortality, without evidence for a role of hydraulic safety risks.

### Outlook

Because our study was carried out in a plantation trial with multiple exotic conifer species, it remains to be tested whether similar drivers and feedback loops operate for conifers within their natural distribution range to which they are better adapted. Moreover, it remains to be tested whether and how these proposed legacy effects apply to angiosperm trees in forests where large species ranges in *LLS* occur. We could expect positive effects of long *LLS* on drought- and heat wave–induced mortality in, for example, tropical moist forests where long *LLS*s reflect adaptation to shade (*46*), not drought or heat. In such forests, evergreen tree species faced higher mortality after drought compared to deciduous species (*47*). In contrast to our conifers, however, hydraulic limitations may also contribute to drought-induced mortality in such tropical moist forests (*48*). For more arid forests, such as Mediterranean forests, dry tropical forests, and tropical savannahs, we expect leaf legacy effects to be smaller since long-lived leaves are adapted to drought (*49*–*52*). In a tropical dry forest, *HSM*s—and not *LLS*—explained species differences in drought-induced tree mortality (*21*, *49*). In a dry tropical savannah in China (*10*), however, drought-induced crown dieback depended on both *HSM*s and *LLS*. The relative roles of different factors and the cascading mechanisms contributing to drought-induced mortality (see Fig. 1) are thus context-dependent and differ across forests. We call for studies on tree mortality that integrate the leaf and growth legacies with hydraulic traits to test the proposed hypotheses and their interdependencies for different forests and climatic regions. Such studies are required to unravel the importance of ecological memory at different time scales, increase our understanding of tree responses to climate change (*23*), and improve tree and forest model predictions of tree mortality and forest dieback in response to extreme climate events (*22*, *53*, *54*).

## MATERIALS AND METHODS

### Experimental design

We measured trees in a common garden experiment situated in the Schovenhorst Estate in Putten, Netherlands (52.25°N, 5.63°E), approximately 30 m above sea level. This site is characterized by an average, annual mean temperature of ~10°C, and average annual rainfall of ~830 mm. Precipitation is quite evenly distributed across seasons. Soils are sandy, well-drained, acidic podzolic soils of low fertility (*55*), with a water table at more than 10 m depth out of reach of tree roots (*56*). Between 1916 and 1974, conifer tree species from across the Northern Hemisphere were planted in small monospecific stands in a relatively small area with maximum distances between these stands <2 km (*57*) from one another, minimizing environmental variation across the species. Trees from the same species belonged to the same provenance, but unfortunately, information on provenance was not collected in a consistent way or has been lost. In 2016, we selected 20 conifer species from this area with sufficient replicate dominant individuals within monospecific stands (18 to >130 individuals) of each species for this study (table S1). From these species, we sampled stem cores in November/December 2017 to quantify stem growth resilience, measured functional hydraulic and leaf traits from June to August 2018 and scored tree mortality in May 2021. All these attributes were measured for light-exposed canopy trees from the same stand, but not necessarily for the same individuals. Hence, we used a species-based approach in which growth and trait values were averaged per species. We do not consider therefore how intraspecific trait variation may have contributed to intraspecific variation in mortality.

To quantify the growth resilience and its components (resistance and recovery) per species, we selected 10 individuals per species with crowns fully exposed in the upper canopy. These trees had an average stem diameter at breast height (1.3 m above soil surface) of 35.8 cm, ranging from 14.8 to 76.9 cm across species (table S1). Both stem diameter and tree age did not significantly contribute to explaining tree mortality differences across species (see fig. S10), and we consider their possible confounding effect on trait effects measured inferior (Fig. 1). We sampled two stem cores at stem breast height and at perpendicular position across the stem in November and December 2017 and measured tree ring widths (at 0.01-mm precision) as proxies for annual stem growth from 1974 until 2018 (*58*). Tree ring time series were cross-dated to assign a calendar year to each ring using CooRecorder, CDendro, and WinTSAP (v. 9.0, Cybis Electronik and Data AB, Rinntech). Cross-dating was done by first matching the ring-width patterns of individual trees, and then different trees of the same species. The resilience indices were calculated using the R “*pointRes*” package (*59*, *60*), with *resistance* = *TRWt*/*TRW*_*t*−2_; *recovery* = *TRW*_*t*+2_/*TRW_t_*; and *resilience* = *TRW*_*t*+2_/*TRW*_*t−*2_, where *TRWt* is the tree ring width at the drought year *t*, *TRW*_*t*−2_ is the average tree ring of the 2 years before the drought year, and *TRW*_*t*+2_ the average ring width of the two years after the drought year t. Resilience indices were calculated on the basis of tree ring width (*TRW*) rather than a detrended ring index because different detrending methods can produce different values (*26*, *61*) and because possible tree age or size effects on stem growth are small in the time window of 5 years as used for our growth resilience analysis. We identified 11 dry summers between 1974 and 2018 based on two criteria after calculating the standardized precipitation evapotranspiration index (in short *SPEI*), which reflects the water balance, for each summer. *SPEI* was calculated for each month as the precipitation minus the potential evapotranspiration (*PET*) summed over four consecutive summer months (June 1 to September 30) and standardized with a probability function (*62*, *63*). We classified a year as dry if *SPEI* <−1, resulting in 11 dry years between 1970 and 2018 (when excluding 2018; fig. S1). Two years (1983 and 1995) were excluded since they did not show a negative cumulative water deficit whereas the other 9 years (1975, 1976, 1982, 1986, 1989, 1996, 2003, 2006, and 2013) did (fig. S2) [for details (*26*)]. The calculation for the resilience indices during 2 consecutive drought years was based on the resistance in the focal year and the growth during 2 years preceding the first drought year and 2 years following the second drought year, to thus exclusively focus on the effects of the drought year of interest (*26*). For our analyses, we averaged the resistance and recovery values per species, and from those averaged growth resistance and growth recovery estimates, we calculated growth resilience per species. Growth resilience responses are shown for the six droughts from 1986 to 2013 covering responses of all 20 species.

For the measurements of functional plant traits in the summer of 2018, we selected five to six fully exposed trees per species for sampling >1-m-long branches from the same tree populations. We sampled branches from a relatively exposed position at around 6-m height, using a telescopic pole (*28*). From these branch samples, we quantified hydraulic traits including pit diameter aperture (*DPA*) and multiple other pit traits (*32*), as well as twig embolism resistance (*P*_50_), turgor loss point *TLP*, minimum leaf water potential (*P*_*min*_), predawn leaf water potential (*P*_*pre*_), and calculated *HSM*s (*HSM* = *P*_*min*_ − *P*_50_) (*28*). We measured *P*_50_ with the standard CAVITRON method (*32*, *64*) and we estimated *P*_*min*_ from the twig water potentials measured between 12:30 and 15:30 from 18 to 25 July and 14 to 19 August, thus including the driest month (July) of 2018 (fig. S2). *P*_*min*_ (as well as *P*_*pre*_, measured before dawn on the same days) was measured for six leaves per species, each leaf measured on a different day. For *P*_*min*_, the lowest values were observed in July, corresponding with the greatest water deficits observed in 2018, and those lowest values were selected as the species-specific *P*_*min*_. *LLS* was estimated by selecting the branch stem segment supporting the most proximal, oldest, needle, and the number of annual rings in the cross section of that segment was counted since it indicates the maximum needle age in years on the branch. Maximum needle ages were then averaged across all sampled branches to get a proxy for *LLS* per species. The *SLA*, *LTD*, and leaf dry matter content (*LDMC*) were measured on random samples taken from the five to six harvested branches per species, which were pooled and from which 100 needles were selected. From this sample, fresh mass (*M*_*F*_) was weighted and fresh volume (*V*_*F*_) was determined with the water displacement method. Needles were scanned to estimate their area (*A*_*F*_) using ImageJ v. 1.52a and then dried at 75°C for 72 hours for dry weight (*M*_*D*_). From this, leaf traits were calculated as follows: *SLA* = *A*_*F*_/*M*_*D*_,* LDMC* = *M*_*D*_/*M*_*F*_, and *LTD* = *M*_*D*_/*V*_*F*_. For details on the measurements, see (*28*).

In May 2021, we quantified the mortality rates of all tree species by an inventory of the number of trees in all plots where we selected our trees for this study. We had the number of live trees in the selected stands for all species before our functional trait measurement campaign in the summer of 2018. The same trees were counted again and scored as dead (without any leaves left) or alive in May 2021. From these counts, we estimated the mortality risks from the ratio of the number of dead trees (in 2021) to the number of alive trees (in 2018), i.e., the fraction of trees that died over the 2 to 3 years following the extremely dry and hot summer of 2018 and subsequent summers of 2019 and 2020, which were also drier than average summers (*31*). Such prolonged mortality responses to a lethal drought and heat year like 2018 over multiple years agree with other studies showing mortality responses protracted over months or years after peak drought intensity (*65*).

### Statistical analysis

To explore how functional traits, resilience indices, tree size, or age affect tree mortality, we used logistic regression {*Y* = *e*^(*ax*+*b*)^/[1 + *e*^(*ax*+*b*)^]} using species as replicates (*N* = 20), where *x* indicates species-level resilience indices or trait values, and *Y* indicates species-level tree mortality data. Similarly, multiple logistic regression was used to determine how the three hypothesized predictors (*HSM*, *LLS*, growth resilience) jointly explained tree mortality across tree species (*N* = 20), with values standardized to compare coefficients by subtracting the mean and dividing it by the standard deviation. All analyses were performed on the species level using the R version (v.4.1.2) (*66*).

## Supplementary Materials

### This PDF file includes:

Figs. S1 to S10 Tables S1 to S3 References

## References

[1] A. Dai, Increasing drought under global warming in observations and models. Nat. Clim. Chang 3, 52–58 (2013).

[2] J. Carnicer, M. Coll, M. Ninyerola, X. Pons, G. Sánchez, J. Peñuelas, Widespread crown condition decline, food web disruption, and amplified tree mortality with increased climate change-type drought. Proc. Natl. Acad. Sci. U.S.A. 108, 1474–1478 (2011). 21220333 10.1073/pnas.1010070108 PMC3029725

[3] A. C. Bennett, N. G. McDowell, C. D. Allen, K. J. Anderson-Teixeira, Larger trees suffer most during drought in forests worldwide. Nat. Plants 1, 15139 (2015). 27251391 10.1038/nplants.2015.139

[4] P. J. Van Mantgem, N. L. Stephenson, J. C. Byrne, L. D. Daniels, J. F. Franklin, P. Z. Fulé, M. E. Harmon, A. J. Larson, J. M. Smith, A. H. Taylor, T. T. Veblen, Widespread increase of tree mortality rates in the western United States. Science 323, 521–524 (2009). 19164752 10.1126/science.1165000

[5] C. D. Allen, A. K. Macalady, H. Chenchouni, D. Bachelet, N. McDowell, M. Vennetier, T. Kitzberger, A. Rigling, D. D. Breshears, E. H. Hogg, P. Gonzalez, R. Fensham, Z. Zhang, J. Castro, N. Demidova, J. H. Lim, G. Allard, S. W. Running, A. Semerci, N. Cobb, A global overview of drought and heat-induced tree mortality reveals emerging climate change risks for forests. For. Ecol. Manage. 259, 660–684 (2010).

[6] P. Ciais, M. Reichstein, N. Viovy, A. Granier, J. Ogée, V. Allard, M. Aubinet, N. Buchmann, C. Bernhofer, A. Carrara, F. Chevallier, N. de Noblet, A. D. Friend, P. Friedlingstein, T. Grünwald, B. Heinesch, P. Keronen, A. Knohl, G. Krinner, D. Loustau, G. Manca, G. Matteucci, F. Miglietta, J. M. Ourcival, D. Papale, K. Pilegaard, S. Rambal, G. Seufert, J. F. Soussana, M. J. Sanz, E. D. Schulze, T. Vesala, R. Valentini, Europe-wide reduction in primary productivity caused by the heat and drought in 2003. Nature 437, 529–533 (2005). 16177786 10.1038/nature03972

[7] H. Hartmann, A. Bastos, A. J. das, A. Esquivel-Muelbert, W. M. Hammond, J. Martínez-Vilalta, N. G. McDowell, J. S. Powers, T. A. M. Pugh, K. X. Ruthrof, C. D. Allen, Climate change risks to global forest health: Emergence of unexpected events of elevated tree mortality worldwide. Annu. Rev. Plant Biol. 73, 673–702 (2022). 35231182 10.1146/annurev-arplant-102820-012804

[8] B. Choat, T. J. Brodribb, C. R. Brodersen, R. A. Duursma, R. López, B. E. Medlyn, Triggers of tree mortality under drought. Nature 558, 531–539 (2018). 29950621 10.1038/s41586-018-0240-x

[9] T. J. Brodribb, J. Powers, H. Cochard, B. Choat, Hanging by a thread? Forests and drought. Science 368, 261–266 (2020). 32299945 10.1126/science.aat7631

[10] Y. J. Chen, B. Choat, F. Sterck, P. Maenpuen, M. Katabuchi, S. B. Zhang, K. W. Tomlinson, R. S. Oliveira, Y. J. Zhang, J. X. Shen, K. F. Cao, S. Jansen, Hydraulic prediction of drought-induced plant dieback and top-kill depends on leaf habit and growth form. Ecol. Lett. 24, 2350–2363 (2021). 34409716 10.1111/ele.13856

[11] N. G. McDowell, G. Sapes, A. Pivovaroff, H. D. Adams, C. D. Allen, W. R. L. Anderegg, M. Arend, D. D. Breshears, T. Brodribb, B. Choat, H. Cochard, M. de Cáceres, M. G. de Kauwe, C. Grossiord, W. M. Hammond, H. Hartmann, G. Hoch, A. Kahmen, T. Klein, D. S. Mackay, M. Mantova, J. Martínez-Vilalta, B. E. Medlyn, M. Mencuccini, A. Nardini, R. S. Oliveira, A. Sala, D. T. Tissue, J. M. Torres-Ruiz, A. M. Trowbridge, A. T. Trugman, E. Wiley, C. Xu, Mechanisms of woody-plant mortality under rising drought, CO_2_ and vapour pressure deficit. Nat. Rev. Earth Environ. 3, 294–308 (2022).

[12] N. McDowell, W. T. Pockman, C. D. Allen, D. D. Breshears, N. Cobb, T. Kolb, J. Plaut, J. Sperry, A. West, D. G. Williams, E. A. Yepez, Mechanisms of plant survival and mortality during drought: Why do some plants survive while others succumb to drought? New Phytol. 178, 719–739 (2008). 18422905 10.1111/j.1469-8137.2008.02436.x

[13] W. M. Hammond, K. Yu, L. A. Wilson, R. E. Will, W. R. L. Anderegg, H. D. Adams, Dead or dying? Quantifying the point of no return from hydraulic failure in drought-induced tree mortality. New Phytol. 223, 1834–1843 (2019). 31087656 10.1111/nph.15922 PMC6771894

[14] C. E. Doughty, D. B. Metcalfe, C. A. J. Girardin, F. F. Amézquita, D. G. Cabrera, W. H. Huasco, J. E. Silva-Espejo, A. Araujo-Murakami, M. C. da Costa, W. Rocha, T. R. Feldpausch, A. L. M. Mendoza, A. C. L. da Costa, P. Meir, O. L. Phillips, Y. Malhi, Drought impact on forest carbon dynamics and fluxes in Amazonia. Nature 519, 78–82 (2015). 25739631 10.1038/nature14213

[15] A. Sala, D. R. Woodruff, F. C. Meinzer, Carbon dynamics in trees: Feast or famine? Tree Physiol. 32, 764–775 (2012). 22302370 10.1093/treephys/tpr143

[16] B. T. Wolfe, Bark water vapour conductance is associated with drought performance in tropical trees. Biol. Lett. 16, 20200263 (2020). 32750268 10.1098/rsbl.2020.0263 PMC7480154

[17] B. T. Wolfe, J. S. Sperry, T. A. Kursar, Does leaf shedding protect stems from cavitation during seasonal droughts? A test of the hydraulic fuse hypothesis. New Phytol. 212, 1007–1018 (2016). 27373446 10.1111/nph.14087

[18] U. Hacke, J. Sperry. (Springer, 2001).

[19] H. D. Adams, M. J. B. Zeppel, W. R. L. Anderegg, H. Hartmann, S. M. Landhäusser, D. T. Tissue, T. E. Huxman, P. J. Hudson, T. E. Franz, C. D. Allen, L. D. L. Anderegg, G. A. Barron-Gafford, D. J. Beerling, D. D. Breshears, T. J. Brodribb, H. Bugmann, R. C. Cobb, A. D. Collins, L. T. Dickman, H. Duan, B. E. Ewers, L. Galiano, D. A. Galvez, N. Garcia-Forner, M. L. Gaylord, M. J. Germino, A. Gessler, U. G. Hacke, R. Hakamada, A. Hector, M. W. Jenkins, J. M. Kane, T. E. Kolb, D. J. Law, J. D. Lewis, J. M. Limousin, D. M. Love, A. K. Macalady, J. Martínez-Vilalta, M. Mencuccini, P. J. Mitchell, J. D. Muss, M. J. O’Brien, A. P. O’Grady, R. E. Pangle, E. A. Pinkard, F. I. Piper, J. A. Plaut, W. T. Pockman, J. Quirk, K. Reinhardt, F. Ripullone, M. G. Ryan, A. Sala, S. Sevanto, J. S. Sperry, R. Vargas, M. Vennetier, D. A. Way, C. Xu, E. A. Yepez, N. G. McDowell, A multi-species synthesis of physiological mechanisms in drought-induced tree mortality. Nat. Ecol. Evol. 1, 1285–1291 (2017). 29046541 10.1038/s41559-017-0248-x

[20] W. R. Anderegg, T. Klein, M. Bartlett, L. Sack, A. F. A. Pellegrini, B. Choat, S. Jansen, Meta-analysis reveals that hydraulic traits explain cross-species patterns of drought-induced tree mortality across the globe. Proc. Natl. Acad. Sci. U.S.A. 113, 5024–5029 (2016). 27091965 10.1073/pnas.1525678113 PMC4983847

[21] J. S. Powers, G. Vargas, T. J. Brodribb, N. B. Schwartz, D. Pérez-Aviles, C. M. Smith-Martin, J. M. Becknell, F. Aureli, R. Blanco, E. Calderón-Morales, J. C. Calvo-Alvarado, A. J. Calvo-Obando, M. M. Chavarría, D. Carvajal-Vanegas, C. D. Jiménez-Rodríguez, E. Murillo Chacon, C. M. Schaffner, L. K. Werden, X. Xu, D. Medvigy, A catastrophic tropical drought kills hydraulically vulnerable tree species. Glob. Chang. Biol. 26, 3122–3133 (2020). 32053250 10.1111/gcb.15037

[22] M. D. Venturas, H. N. Todd, A. T. Trugman, W. R. Anderegg, Understanding and predicting forest mortality in the western United States using long-term forest inventory data and modeled hydraulic damage. New Phytol. 230, 1896–1910 (2021). 33112415 10.1111/nph.17043

[23] R. Zweifel, F. Sterck, A conceptual tree model explaining legacy effects on stem growth. Front. For. Glob. Change 1, 00009 (2018).

[24] N. K. Ruehr, R. Grote, S. Mayr, A. Arneth, Beyond the extreme: Recovery of carbon and water relations in woody plants following heat and drought stress. Tree Physiol. 39, 1285–1299 (2019). 30924906 10.1093/treephys/tpz032 PMC6703153

[25] F. Magnani, M. Mencuccini, J. Grace, Age-related decline in stand productivity: The role of structural acclimation under hydraulic constraints. Plant Cell Environ. 23, 251–263 (2000).

[26] Y. Song, F. Sterck, U. Sass-Klaassen, C. Li, L. Poorter, Growth resilience of conifer species decreases with early, long-lasting and intense droughts but cannot be explained by hydraulic traits. J. Ecol. 110, 2088–2104 (2022).

[27] L. DeSoto, M. Cailleret, F. Sterck, S. Jansen, K. Kramer, E. M. R. Robert, T. Aakala, M. M. Amoroso, C. Bigler, J. J. Camarero, K. Čufar, G. Gea-Izquierdo, S. Gillner, L. J. Haavik, A.-M. Hereş, J. M. Kane, V. I. Kharuk, T. Kitzberger, T. Klein, T. Levanič, J. C. Linares, H. Mäkinen, W. Oberhuber, A. Papadopoulos, B. Rohner, G. Sangüesa-Barreda, D. B. Stojanovic, M. L. Suárez, R. Villalba, J. Martínez-Vilalta, Low growth resilience to drought is related to future mortality risk in trees. Nat. Commun. 11, 545 (2020). 31992718 10.1038/s41467-020-14300-5 PMC6987235

[28] Y. Song, F. Sterck, X. Zhou, Q. Liu, B. Kruijt, L. Poorter, Drought resilience of conifer species is driven by leaf lifespan but not by hydraulic traits. New Phytol. 235, 978–992 (2022). 35474217 10.1111/nph.18177 PMC9322575

[29] L. Plavcová, G. Hoch, H. Morris, S. Ghiasi, S. Jansen, The amount of parenchyma and living fibers affects storage of nonstructural carbohydrates in young stems and roots of temperate trees. Am. J. Bot. 103, 603–612 (2016). 26993972 10.3732/ajb.1500489

[30] V. B. Möhring, A. Bitter, G. Bub, M. Dieter, M. Dög, M. Hanewinkel, N. G. von Hatzfeldt, J. Köhler, G. Ontrup, R. Rosenberger, B. Seintsch, F. Thoma, Schadenssumme insgesamt 12,7 Mrd. Euro: Abschätzung der ökonomischen Schäden der Extremwetterereignisse der Jahre 2018 bis 2020 in der Forstwirtschaft. Forstwirtschaft 9, 155–158 (2021).

[31] W. Peters, A. Bastos, P. Ciais, A. Vermeulen, A historical, geographical and ecological perspective on the 2018 European summer drought. Philos. Trans. R. Soc. B 375, 20190505 (2020). 10.1098/rstb.2019.0505 PMC7485105 32892723

[32] Y. Song, L. Poorter, A. Horsting, S. Delzon, F. Sterck, Pit and tracheid anatomy explain hydraulic safety but not hydraulic efficiency of 28 conifer species. J. Exp. Bot. 73, 1033–1048 (2022). 34626106 10.1093/jxb/erab449 PMC8793876

[33] B. Choat, S. Jansen, T. J. Brodribb, H. Cochard, S. Delzon, R. Bhaskar, S. J. Bucci, T. S. Feild, S. M. Gleason, U. G. Hacke, A. L. Jacobsen, F. Lens, H. Maherali, J. Martínez-Vilalta, S. Mayr, M. Mencuccini, P. J. Mitchell, A. Nardini, J. Pittermann, R. B. Pratt, J. S. Sperry, M. Westoby, I. J. Wright, A. E. Zanne, Global convergence in the vulnerability of forests to drought. Nature 491, 752–755 (2012). 23172141 10.1038/nature11688

[34] J. Martínez-Vilalta, A. Sala, J. Piñol, The hydraulic architecture of Pinaceae–a review. Plant Ecol. 171, 3–13 (2004).

[35] D. M. Johnson, K. A. McCulloh, D. R. Woodruff, F. C. Meinzer, Hydraulic safety margins and embolism reversal in stems and leaves: Why are conifers and angiosperms so different? Plant Sci. 195, 48–53 (2012). 22920998 10.1016/j.plantsci.2012.06.010

[36] C. Körner, No need for pipes when the well is dry—a comment on hydraulic failure in trees. Tree Physiol. 39, 695–700 (2019). 30938423 10.1093/treephys/tpz030

[37] M. Arend, R. M. Link, R. Patthey, G. Hoch, B. Schuldt, A. Kahmen, Rapid hydraulic collapse as cause of drought-induced mortality in conifers. Proc. Natl. Acad. Sci. U.S.A. 118, e2025251118 (2021). 33846261 10.1073/pnas.2025251118 PMC8072240

[38] W. Wang, B. Vinocur, A. Altman, Plant responses to drought, salinity and extreme temperatures: Towards genetic engineering for stress tolerance. Planta 218, 1–14 (2003). 14513379 10.1007/s00425-003-1105-5

[39] R. Teskey, T. Wertin, I. Bauweraerts, M. Ameye, M. A. M. Guire, K. Steppe, Responses of tree species to heat waves and extreme heat events. Plant Cell Environ. 38, 1699–1712 (2015). 25065257 10.1111/pce.12417

[40] P. Krokene, Conifer Defense and Resistance to Bark Beetles, in *Bark Beetles* (Elsevier, 2015), pp. 177–207.

[41] P. Reich, M. Walters, B. Kloeppel, D. Ellsworth, Different photosynthesis-nitrogen relations in deciduous hardwood and evergreen coniferous tree species. Oecologia 104, 24–30 (1995). 28306909 10.1007/BF00365558

[42] C. Fortunel, C. Stahl, S. Coste, C. Ziegler, G. Derroire, S. Levionnois, I. Maréchaux, D. Bonal, B. Hérault, F. H. Wagner, L. Sack, J. Chave, P. Heuret, S. Jansen, G. John, C. Scoffoni, S. Trueba, M. K. Bartlett, Thresholds for persistent leaf photochemical damage predict plant drought resilience in a tropical rainforest. New Phytol. 239, 576–591 (2023). 37222272 10.1111/nph.18973

[43] J. Margalef-Marrase, M. Á. Pérez-Navarro, F. Lloret, Relationship between heatwave-induced forest die-off and climatic suitability in multiple tree species. Glob. Chang. Biol. 26, 3134–3146 (2020). 32064733 10.1111/gcb.15042

[44] J. A. Sohn, S. Saha, J. Bauhus, Potential of forest thinning to mitigate drought stress: A meta-analysis. For. Ecol. Manage. 380, 261–273 (2016).

[45] T. J. Brodribb, D. J. Bowman, S. Nichols, S. Delzon, R. Burlett, Xylem function and growth rate interact to determine recovery rates after exposure to extreme water deficit. New Phytol. 188, 533–542 (2010). 20673281 10.1111/j.1469-8137.2010.03393.x

[46] F. Sterck, L. Poorter, F. Schieving, Leaf traits determine the growth-survival trade-off across rain forest tree species. Am. Nat. 167, 758–765 (2006). 16671019 10.1086/503056

[47] I. Aleixo, D. Norris, L. Hemerik, A. Barbosa, E. Prata, F. Costa, L. Poorter, Amazonian rainforest tree mortality driven by climate and functional traits. Nat. Clim. Chang. 9, 384–388 (2019).

[48] L. Rowland, A. C. L. da Costa, D. R. Galbraith, R. S. Oliveira, O. J. Binks, A. A. R. Oliveira, A. M. Pullen, C. E. Doughty, D. B. Metcalfe, S. S. Vasconcelos, L. V. Ferreira, Y. Malhi, J. Grace, M. Mencuccini, P. Meir, Death from drought in tropical forests is triggered by hydraulics not carbon starvation. Nature 528, 119–122 (2015). 26595275 10.1038/nature15539

[49] R. S. Oliveira, C. B. Eller, F. V. Barros, M. Hirota, M. Brum, P. Bittencourt, Linking plant hydraulics and the fast–slow continuum to understand resilience to drought in tropical ecosystems. New Phytol. 230, 904–923 (2021). 33570772 10.1111/nph.17266

[50] D. Ackerly, Functional strategies of chaparral shrubs in relation to seasonal water deficit and disturbance. Ecol. Monogr. 74, 25–44 (2004).

[51] J. L. Quero, F. J. Sterck, J. Martínez-Vilalta, R. Villar, Water-use strategies of six co-existing Mediterranean woody species during a summer drought. Oecologia 166, 45–57 (2011). 21290148 10.1007/s00442-011-1922-3

[52] A. L. Pivovaroff, S. C. Pasquini, M. E. de Guzman, K. P. Alstad, J. S. Stemke, L. S. Santiago, Multiple strategies for drought survival among woody plant species. Funct. Ecol. 30, 517–526 (2016).

[53] A. T. Trugman, L. D. Anderegg, W. R. Anderegg, A. J. Das, N. L. Stephenson, Why is tree drought mortality so hard to predict? Trends Ecol. Evol. 36, 520–532 (2021). 33674131 10.1016/j.tree.2021.02.001

[54] L. Rowland, J. Martínez-Vilalta, M. Mencuccini, Hard times for high expectations from hydraulics: Predicting drought-induced forest mortality at landscape scales remains a challenge. New Phytol. 230, 1685–1687 (2021). 33797779 10.1111/nph.17317

[55] H. Cornelissen, U. Sass-Klaassen, L. Poorter, K. van Geffen, R. S. P. van Logtestijn, J. van Hal, L. Goudzwaard, F. J. Sterck, R. K. W. M. Klaassen, G. T. Freschet, A. van der Wal, H. Eshuis, J. Zuo, W. de Boer, T. Lamers, M. Weemstra, V. Cretin, R. Martin, J. den Ouden, M. P. Berg, R. Aerts, G. M. J. Mohren, M. M. Hefting, Controls on coarse wood decay in temperate tree species: Birth of the LOGLIFE experiment. Ambio 41, 231–245 (2012). 22864697 10.1007/s13280-012-0304-3 PMC3535053

[56] TNO-NITG, www.cinoloket.nl [accessed January 2020].

[57] J. J. H. Willinge Gratama-Oudemans, The arboretum of Schovenhorst, Putten, in the Netherlands. Arboric. J. 16, 197–205 (1992).

[58] Y. Song, U. Sass-Klaassen, F. Sterck, L. Goudzwaard, L. Akhmetzyanov, L. Poorter, Growth of 19 conifer species is highly sensitive to winter warming, spring frost and summer drought. Ann. Bot. 128, 545–557 (2021). 34216460 10.1093/aob/mcab090 PMC8422889

[59] V. Vitali, U. Büntgen, J. Bauhus, Silver fir and Douglas fir are more tolerant to extreme droughts than Norway spruce in south-western Germany. Glob. Chang. Biol. 23, 5108–5119 (2017). 28556403 10.1111/gcb.13774

[60] M. van der Maaten-Theunissen, E. van der Maaten, O. Bouriaud, pointRes: An R package to analyze pointer years and components of resilience. Dendrochronologia 35, 34–38 (2015).

[61] R. L. Peters, P. Groenendijk, M. Vlam, P. A. Zuidema, Detecting long-term growth trends using tree rings: A critical evaluation of methods. Glob. Chang. Biol. 21, 2040–2054 (2015). 25482401 10.1111/gcb.12826

[62] S. M. Vicente-Serrano, S. Beguería, J. I. López-Moreno, A multiscalar drought index sensitive to global warming: The standardized precipitation evapotranspiration index. J. Clim. 23, 1696–1718 (2010).

[63] S. Beguería, S. Vicente-Serrano, *Calculation of the Standardised Precipitation-Evapotranspiration Index* (2013); SPEI R package version 1.

[64] S. Delzon, C. Douthe, A. Sala, H. Cochard, Mechanism of water-stress induced cavitation in conifers: Bordered pit structure and function support the hypothesis of seal capillary-seeding. Plant Cell Environ. 33, 2101–2111 (2010). 20636490 10.1111/j.1365-3040.2010.02208.x PMC3003904

[65] X. Wu, H. Liu, X. Li, P. Ciais, F. Babst, W. Guo, C. Zhang, V. Magliulo, M. Pavelka, S. Liu, Y. Huang, P. Wang, C. Shi, Y. Ma, Differentiating drought legacy effects on vegetation growth over the temperate Northern Hemisphere. Glob. Chang. Biol. 24, 504–516 (2018). 28973825 10.1111/gcb.13920

[66] R Core Team, *R: A Language and Environment for Statistical Computing* (R Foundation for Statistical Computing, 2021) version 4.1. 2; www.R-project.org/.

[67] M. Weemstra, B. Eilmann, U. G. Sass-Klaassen, F. J. Sterck, Summer droughts limit treegrowth across 10 temperate species on a productive forest site. For. Ecol. Manage. 306, 142–149 (2013).

[68] A. Farjon, D. Filer, An Atlas of the World’s Conifers: An Analysis of Their Distribution, Biogeography, Diversity and Conservation Status. (Brill, 2013).

