# Supplementary material for: Drought- and heat-induced mortality of conifer trees is explained by leaf and growth legacies
Source: Sci Adv. 2024 Apr 12;10(15):eadl4800. doi: 10.1126/sciadv.adl4800 (PMC11014445; doi:10.1126/sciadv.adl4800)
Supplement: Supplementary file 1 — Figs. S1 to S10 Tables S1 to S3 References [file sciadv.adl4800_sm.pdf]

Science Advances
AAAS

Supplementary Materials for

Drought- and heat-induced mortality of conifer trees is explained by leaf and growth legacies

Frank J. Sterck *et al.*

Corresponding author: Frank J. Sterck, frank.sterck@wur.nlan; Yanjun Song, yanjun.song2021@gmail.com

Sci. Adv. **10**, eadl4800 (2024)
DOI: 10.1126/sciadv.adl4800

This PDF file includes:

Figs. S1 to S10
Tables S1 to S3
References

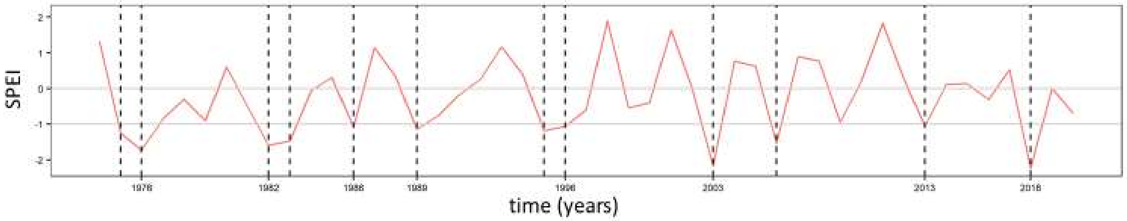

Fig. S1. Proxies for annual drought were quantified from 1970 to 2020 using the Standardized Precipitation Evapotranspiration Index, in short SPEI, following Vicente-Serrano et al. 2010*(62)* and Beguería et al. 2013*(66)*. SPEI has an average of zero and standard deviation of 1 and was calculated for 4-month summer periods (June-July-August-September) to cover the major summer droughts in the Dutch climate*(67)*, using the R 'SPEI' package*(66)*. Years with gridlines were characterized by SPEI<-1 and considered drought years. We thus detected 11 dry summers between 1970 and 2018 (with 2018 excluded). From this, the six drought years from 1986-2013 were selected for quantifying growth resilience to drought (see for explanation, methods).

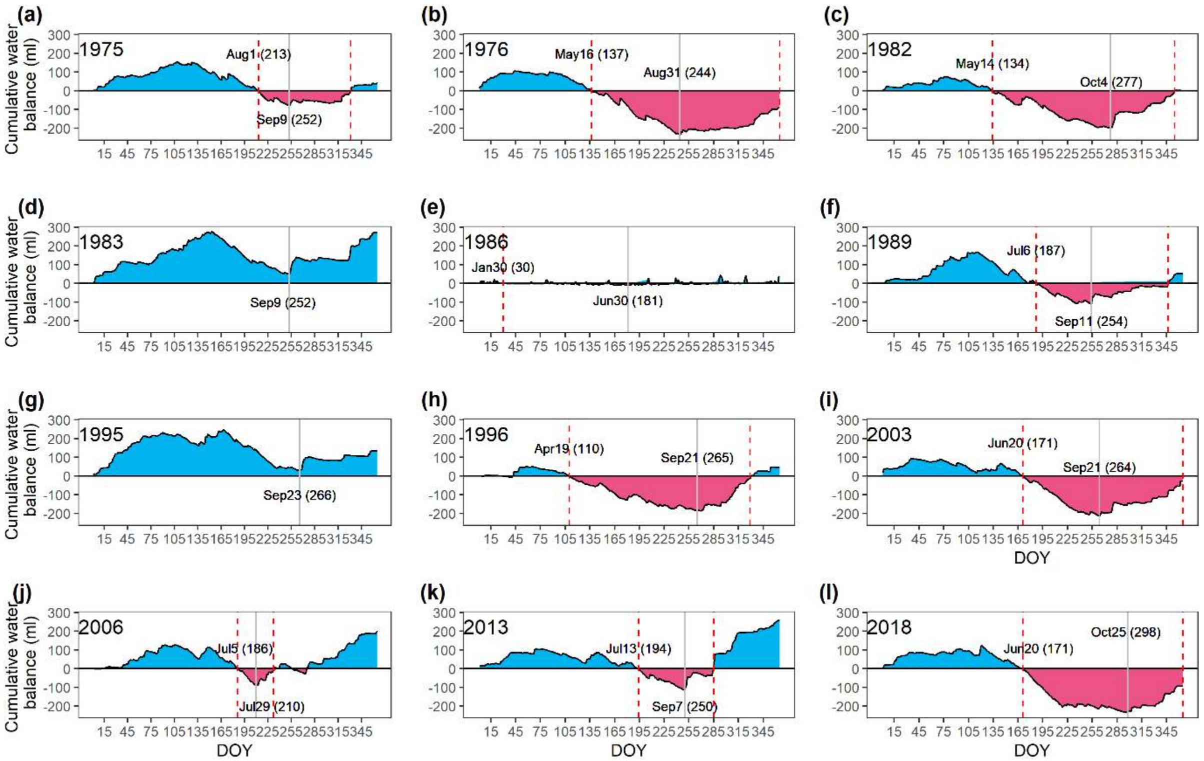

Fig. S2. Annual development of the cumulative water balance visualised for 12 years with the Standardized Precipitation Evapotranspiration Index SPEI<-1 , including 2018 (see Fig. S1). Periods with surplus of water CWB>0 (blue area) and cumulative water deficit DWB<0 (red area) are visualised. This cumulative water deficit was calculated by summing the daily water balance in the period January 1-December 31 of each year. Daily water balance was calculated as the difference between daily precipitation and daily evaporation with climate local data: https://www.knmi.nl/nederland-nu/klimatologie/daggegevens). DOY stands for the Day number Of the Year. The start and end of the dry period are indicated with dashed, red, gridlines, the day with the lowest value with a grey line.

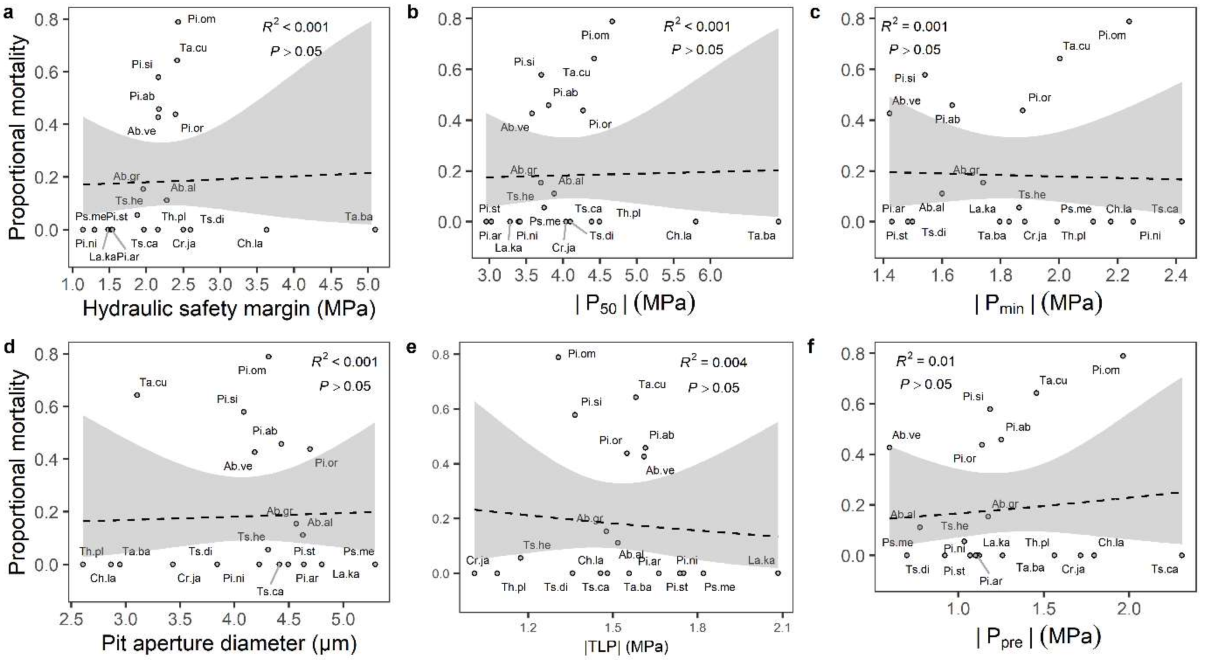

Fig. S3. Patterns of mortality rates versus hydraulic traits across 20 conifer species. Predictions from logistic models are non-significant (P>0.05) and therefore shown by dashed lines, not solid lines. For hydraulic trait abbreviations, see Fig. 1; for species abbreviations, see Table S1.

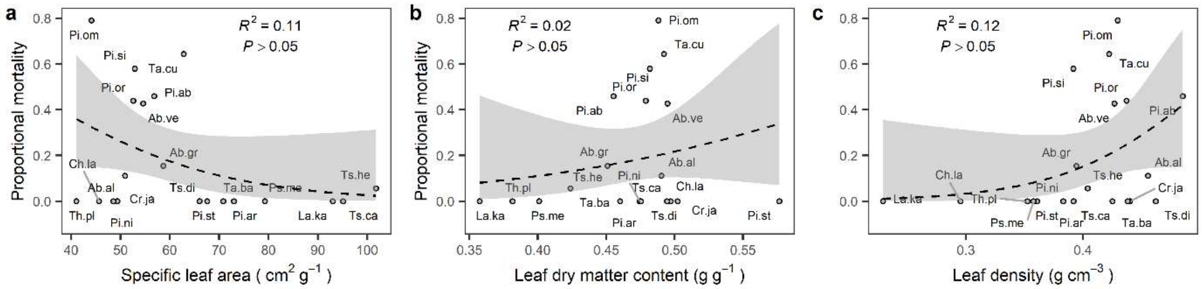

Fig. S4. Patterns of mortality rates in relation to various leaf traits across 20 conifer species. Predictions from logistic models are shown by solid lines if significant ($P \leq 0.05$), or dashed lines if not ($P > 0.05$).

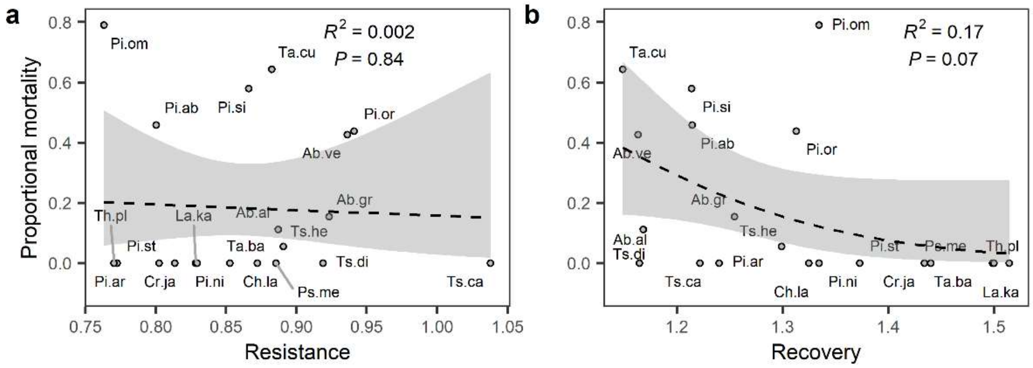

Fig. S5. Patterns of mortality rates versus two resilience components across 20 conifer species. Predictions from logistic models are shown by solid lines if significant ($P \leq 0.05$), or dashed lines if not ($P > 0.05$).

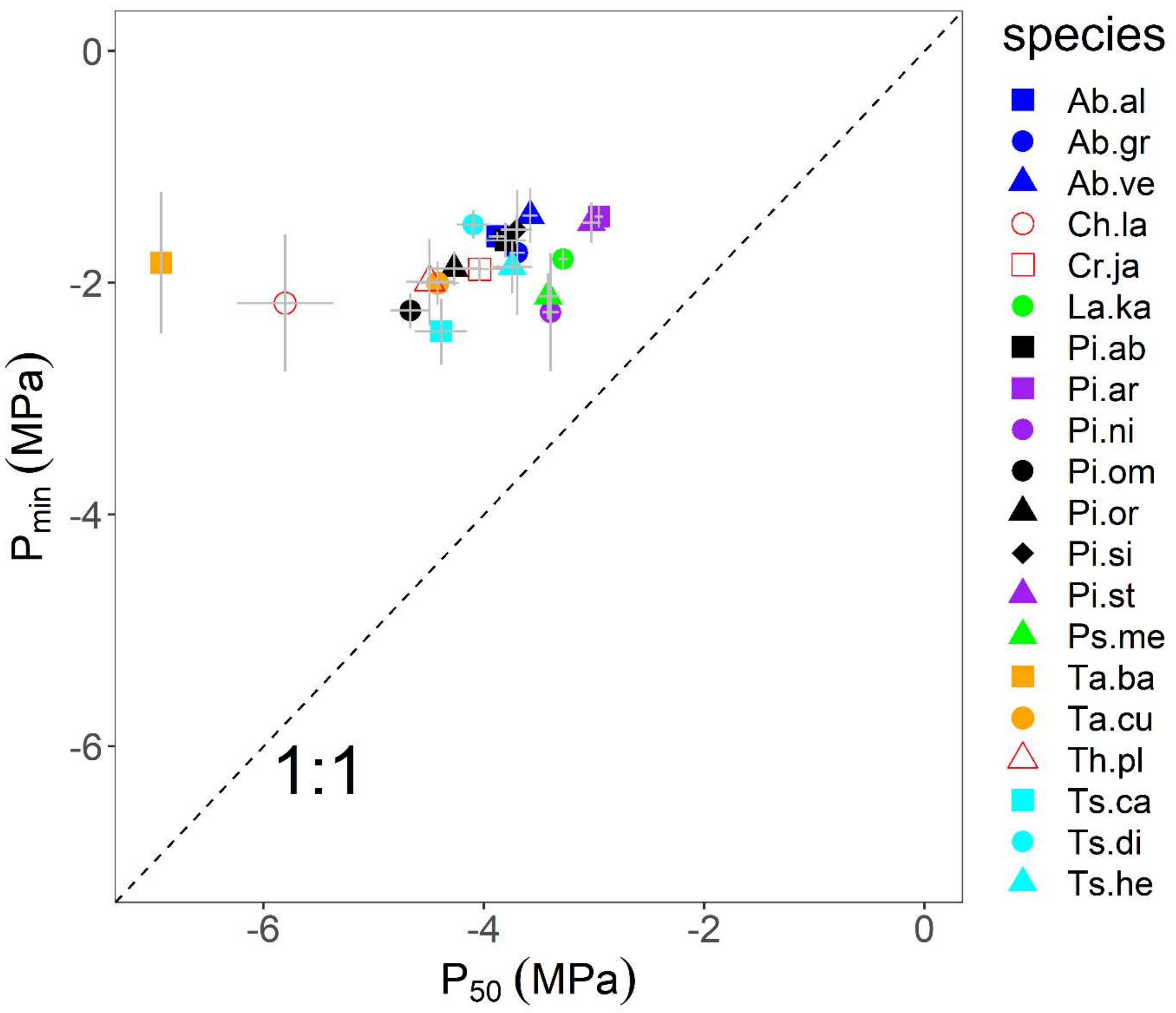

Fig. S6. The minimum water potential during the growing season as measured for shoots (P_{min}) versus the embolism resistance (P_{50}). The hydraulic safety margin (HSM) is the vertical distance between the points and the 1:1 dashed, diagonal, line. For species abbreviations, see Table S1. Errors bars represent 95% confidence intervals of the mean.

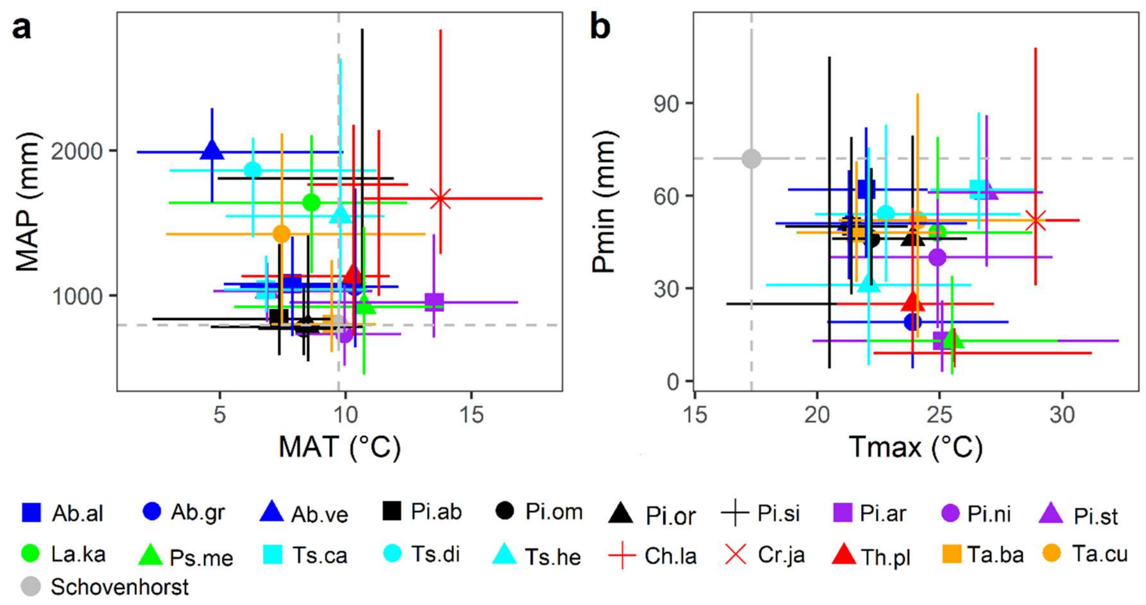

Fig. S7. Comparison of critical niche borders for high temperature and low rainfall across the 20 conifer tree species studied. For species codes, see Table S1. MAT and Tmax stand for the mean annual temperature and the maximum monthly temperature within the geographic distribution range of each species respectively, and MAP and Pmin for mean annual precipitation and minimum monthly precipitation respectively. The symbols indicate the 50% quantile values and the error bars the range from 10% to 90% quantiles. The gridlines indicate the conditions for the common garden study site at the Schovenhorst Estate forest, the Netherlands. The climate distribution ranges of species were obtained from the Global Biodiversity Information Facility GBIF: https://gbif.org/ (for details, Song et al. 2022 *(28)*).

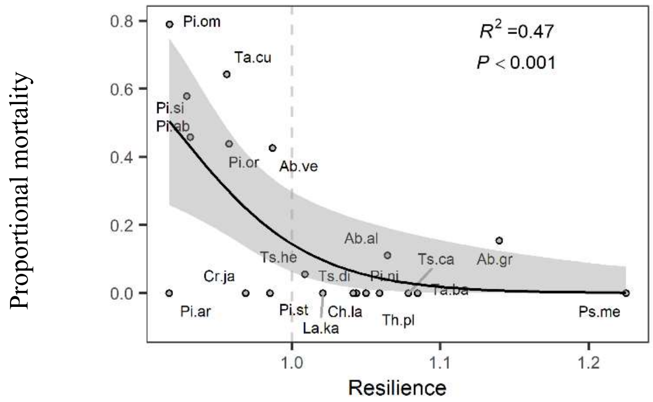

Fig. S8. Conifer species comparisons for patterns of mortality in response to the 2018 drought year with growth resilience to 8 dry periods preceding the 2018 drought, thus adding the 1975-1976 and 1982-1983 droughts to the series from 1986 (see Figure 2c). This longer time series is equal to the one used in Song et al. 2022 *(26)* for the same set of species, but the design was not fully balanced since these two early droughts were not included for *Pinus armandii*, and the 1975-1976 drought not for *Abies veichii* (Table S1). The results were nevertheless similar, showing a significant decrease in mortality with increased growth resilience to droughts preceding 2018.

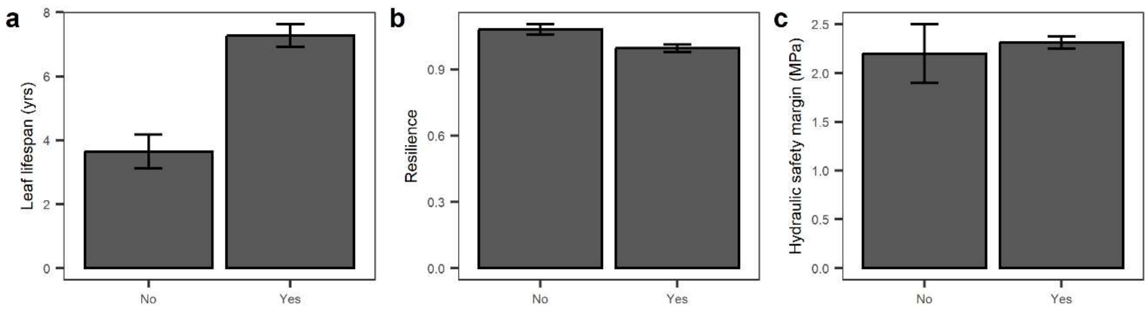

Fig. S9. Relationships between the presence of bark beetle exit holes on tree stems (on x-axis: no, yes) for different species (see Table S1), an important pest for many conifers, and the investigated three factors: a) leaf life span (Student T-Test T=-5.66, P<0.01), b) Resilience (Student T-Test T=-2.89, P=0.01), and c) hydraulic safety margin (Student T-Test T=-0.37, P=0.72) for the 20 tree species (N=20). Bars represent species averages and standard errors are added.

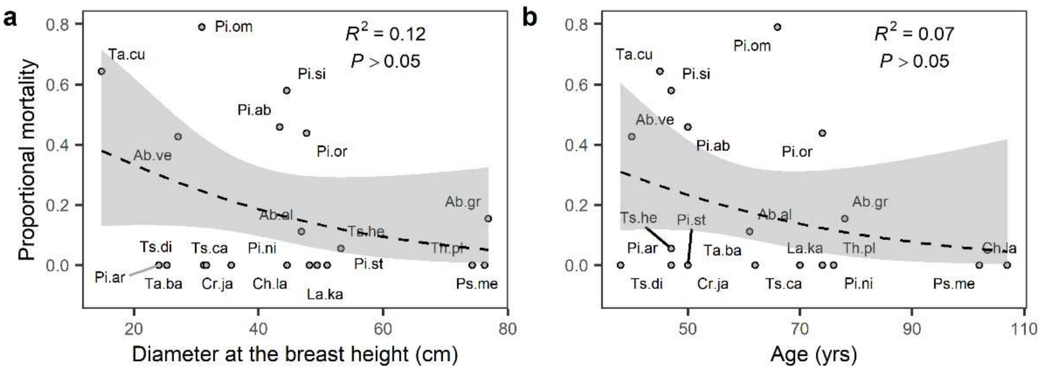

Fig. S10. Patterns of mortality rates versus tree size or age across 20 conifer species. The predictions from logistic models were non-significant and are therefore shown as dashed lines (*P*≤0.05), Species abbreviations are explained in Table S1.

Table S1. Overview of 20 conifer species, their species name, abbreviation code (as used in some Figures), original regional distribution, the period over which tree rings were analyzed, and the average stem diameter at breast height (DBH) of trees sampled for stem ring and traits measurements (N=10), and its standard deviation (in parentheses). Distribution areas are obtained from Farjon and Filer*(68)*. Beetle presence was checked based on the presence of exit holes in trees in 2021. Further, we show species specific scores of tree mortality (MR) and of a number of tree rings statistics (see for details on growth resilience methods and results on these species Song et al. 2022 *(26)*: average tree ring width (TRW), Rbar which indicates inter tree ring series autocorrelation, EPS which indicates expressed population signals and MS which indicates sensitivity.

**Species**	**code**	**Distribution**	**Period**	**DBH (cm)**	**Beetle**	**MR**	**TRW**	**Rbar**	**EPS**	**MS**
Abies alba	Ab.al	Europe	1958-2018	46.9 (10.3)	Yes[1]	0.11	4.7	0.24	0.79	0.28
Abies grandis	Ab.gr	North America	1940-2017	76.9 (8.7)	No	0.15	4.4	0.24	0.82	0.28
Abies veitchii	Ab.ve	North Honshu, Japan	1979-2018	27.1 (3.1)	Yes[1]	0.43	3.0	0.41	0.88	0.39
Chamaecyparis lawsoniana	Ch.la	North America (USA)	1911-2017	48.2 (9.6)	No	0.00	3.2	0.34	0.81	0.36
Cryptomeria japonica	Cr.ja	Eastern Asia	1969-2018	35.6 (6.2)	No	0.00	3.2	0.44	0.90	0.41
Larix kaempferi	La.ka	Eastern Asia	1945-2018	49.4 (6.8)	No	0.00	2.9	0.61	0.95	0.45
Pinus armandii	Pi.ar	Eastern Asia	1981-2018	24.0 (3.2)	No	0.00	3.0	0.32	0.87	0.26
Pinus nigra	Pi.ni	South-East Europe	1945-2018	44.6 (6.5)	No	0.00	2.4	0.30	0.87	0.26
Pinus strobus	Pi.st	North America	1971-2020	51.0 (7.0)	No	0.00	4.8	0.62	0.80	0.27
Picea abies	Pi.ab	Europe	1969-2018	43.4 (5.2)	Yes	0.46	4.0	0.43	0.91	0.28
Picea omorika	Pi.om	Europe	1953-2018	30.9 (3.1)	Yes	0.79	2.8	0.32	0.81	0.30
Picea orientalis	Pi.or	Mainland Asia	1944-2017	47.7 (8.1)	Yes	0.44	3.0	0.46	0.92	0.30
Picea sitchensis	Pi.si	North America	1972-2018	44.5 (8.8)	Yes	0.58	4.7	0.28	0.85	0.22
Pseudotsuga menziesii	Ps.me	North America	1916-2017	76.3 (10.1)	No	0.00	3.7	0.40	0.87	0.31
Taxus baccata	Ta.ba	Europe	1957-2018	31.2 (9.9)	No	0.00	2.2	0.48	0.90	0.28
Taxus cuspidata	Ta.cu	Mainland Asia	1974-2018	14.8 (4.0)	No	0.64	1.6	0.34	0.83	0.25
Thuja plicata	Th.pl	North America	1942-2017	74.3 (12.7)	No	0.00	4.0	0.33	0.86	0.41
Tsuga canadensis	Ts.ca	North America	1951-2020	31.6 (4.8)	No	0.00	1.8	0.56	0.81	0.31
Tsuga diversifolia	Ts.di	Japan	1972-2018	25.3 (3.6)	Yes[1]	0.00	2.7	0.44	0.88	0.28
Tsuga heterophylla	Ts.he	North America	1971-2017	53.2 (6.4)	No	0.06	4.9	0.28	0.85	0.28

[1] only <10% individuals with exit holes in stems

Table S2. Correlation coefficients for relationships between mortality, growth resilience and functional plant traits across 20 conifer species at Schovenhorst, the Netherlands, are shown below the diagonal, and phylogenetic independent contrast correlations are shown above the diagonal. Spearman correlations coefficients are shown for all correlations with tree mortality (MR, not normally distributed), and Pearson correlation coefficients for all others (all normally distributed). MR = mortality, R1= growth resistance, R2 = stem recovery, R3 = resilience, HSM = hydraulic safety margin calculated as P_{min}-P_{50}, HSM* = hydraulic safety margin calculated as TLP-P_{50}, $|P_{50}|$ = embolism resistance as water potential with 50% water conductance loss in branch segment (in MPa), |TLP| = turgor loss point, $|P_{min}|$= midday twig water potentials (in MPa), $|P_{pre}|$ = predawn twig water potential (in MPa), DPA = pit aperture diameter (in μm), WD = wood density (in cm^3 g^{-1}), LLS = leaf lifespan (in yrs), LTD = Leaf tissue density (in cm^3 g^{-1}), LDMC = leaf dry matter content (in g g^{-1}) and SLA = specific leaf area (in cm^2 g^{-1}). The bold and underlined correlations indicate *P*<0.01 and bold correlations indicate 0.01≤*P*<0.05.

	MR	R1	R2	R3	HSM	HSM*	$\|P_{50}\|$	|TLP|	$\|P_{min}\|$	$\|P_{pre}\|$	DPA	WD	LLS	LTD	LDMC	SLA
MR		0.06	-0.31	**-0.62**	0.04	-0.11	-0.09	-0.25	0.00	0.13	-0.02	-0.06	**0.47**	-0.05	0.13	-0.22
R1	0.11		-0.32	0.33	-0.04	-0.03	0.13	**0.60**	**0.49**	**0.51**	0.25	-0.26	0.03	0.00	**0.54**	0.02
R2	**-0.54**	-0.44		**0.68**	**0.62**	**0.72**	**0.64**	-0.08	-0.12	-0.15	-0.22	0.21	**-0.78**	-0.15	-0.42	0.24
R3	**-0.55**	0.44	**0.48**		**0.56**	**0.70**	**0.74**	0.37	0.31	0.28	0.15	0.02	**-0.65**	-0.01	0.09	0.21
HSM	0.25	0.09	0.01	-0.02		**0.94**	**0.92**	0.02	-0.20	-0.05	**-0.46**	**0.54**	-0.42	0.17	-0.01	0.02
HSM*	0.06	0.07	0.20	0.19	**0.91**		**0.98**	-0.02	0.06	0.12	-0.43	0.43	**-0.50**	0.11	-0.02	0.01
$\|P_{50}\|$	0.17	0.14	0.18	0.23	**0.90**	**0.98**		0.12	0.18	0.23	-0.35	0.38	**-0.46**	0.09	0.05	0.01
|TLP|	-0.16	0.04	0.06	0.18	-0.42	**-0.51**	-0.37		0.22	0.20	**0.47**	-0.21	-0.26	-0.20	0.17	0.44
$\|P_{min}\|$	-0.10	0.15	0.31	0.44	0.00	0.35	0.41	-0.10		**0.82**	0.23	-0.45	0.03	-0.20	0.30	-0.11
$\|P_{pre}\|$	0.08	-0.01	0.14	0.04	0.30	**0.49**	**0.48**	-0.40	**0.65**		0.13	-0.40	-0.07	-0.01	0.45	-0.08
DPA	0.05	0.19	-0.16	0.14	**-0.70**	**-0.75**	**-0.70**	**0.53**	-0.17	-0.41		**-0.52**	0.11	-0.03	0.28	0.13
WD	0.29	0.14	-0.25	-0.19	**0.52**	**0.45**	**0.46**	-0.24	-0.06	0.07	-0.25		-0.05	0.11	-0.27	0.00
LLS	**0.84**	0.25	**-0.66**	-0.39	0.34	0.21	0.23	-0.31	-0.10	-0.02	-0.05	0.42		0.25	0.35	**-0.52**
LTD	0.37	0.19	**-0.54**	-0.23	0.29	0.21	0.15	-0.42	-0.24	-0.03	0.00	0.42	**0.64**		0.36	-0.32
LDMC	0.11	0.11	**-0.46**	-0.18	0.14	0.06	0.03	-0.12	-0.17	0.16	-0.05	-0.01	0.35	**0.46**		-0.21
SLA	-0.25	0.36	0.09	0.40	-0.28	-0.25	-0.23	0.33	0.03	-0.07	0.39	0.05	-0.42	-0.20	-0.30	

Table S3. Test statistics (regression coefficients and probability values) of a multiple logistic regression model predicting the mortality risks from the hydraulic safety margin (HSM) , leaf lifespan and growth resilience (Resilience) across 20 conifer tree species. To account for possible collinearity between the predictors leaf life span and resilience, we replaced growth resilience with the residuals of growth resilience against leaf life span using a linear regression model: the results were very similar to the results presented in Table 2, and confirm that leaf life span and growth resilience have at least partially additive effects in explaining tree mortality.

	Independent variables			Intercept	R^2
	HSM	Leaf lifespan	Residuals (Resilience vs lifespan)		
Coefficients	-0.39	2.29	-1.08	-2.95	0.84
P-values	0.71	<0.001	0.02	<0.001	<0.001

REFERENCES AND NOTES

1. A. Dai, Increasing drought under global warming in observations and models. *Nat. Clim. Change.* **3**, 52–58 (2013).

2. J. Carnicer, M. Coll, M. Ninyerola, X. Pons, G. Sánchez, J. Peñuelas, Widespread crown condition decline, food web disruption, and amplified tree mortality with increased climate change-type drought. *Proc. Natl. Acad. Sci.U.S.A.* **108**, 1474–1478 (2011).

3. A. C. Bennett, N. G. McDowell, C. D. Allen, K. J. Anderson-Teixeira, Larger trees suffer most during drought in forests worldwide. *Nat. Plants.* **1**, 15139 (2015).

4. P. J. Van Mantgem, N. L. Stephenson, J. C. Byrne, L. D. Daniels, J. F. Franklin, P. Z. Fulé, M. E. Harmon, A. J. Larson, J. M. Smith, A. H. Taylor, T. T. Veblen, Widespread increase of tree mortality rates in the western United States. *Science* **323**, 521–524 (2009).

5. C. D. Allen, A. K. Macalady, H. Chenchouni, D. Bachelet, N. McDowell, M. Vennetier, T. Kitzberger, A. Rigling, D. D. Breshears, E. H. (T.) Hogg, P. Gonzalez, R. Fensham, Z. Zhang, J. Castro, N. Demidova, J. H. Lim, G. Allard, S. W. Running, A. Semerci, N. Cobb, A global overview of drought and heat-induced tree mortality reveals emerging climate change risks for forests. *For. Ecol. Manage.* **259**, 660–684 (2010).

6. P. Ciais, M. Reichstein, N. Viovy, A. Granier, J. Ogée, V. Allard, M. Aubinet, N. Buchmann, C. Bernhofer, A. Carrara, F. Chevallier, N. de Noblet, A. D. Friend, P. Friedlingstein, T. Grünwald, B. Heinesch, P. Keronen, A. Knohl, G. Krinner, D. Loustau, G. Manca, G. Matteucci, F. Miglietta, J. M. Ourcival, D. Papale, K. Pilegaard, S. Rambal, G. Seufert, J. F. Soussana, M. J. Sanz, E. D. Schulze, T. Vesala, R. Valentini, Europe-wide reduction in primary productivity caused by the heat and drought in 2003. *Nature* **437**, 529–533 (2005).

7. H. Hartmann, A. Bastos, A. J. das, A. Esquivel-Muelbert, W. M. Hammond, J. Martínez-Vilalta, N. G. McDowell, J. S. Powers, T. A. M. Pugh, K. X. Ruthrof, C. D. Allen, Climate change risks to global forest health: Emergence of unexpected events of elevated tree mortality worldwide. *Annu. Rev. Plant Biol.* **73**, 673–702 (2022).

8. B. Choat, T. J. Brodribb, C. R. Brodersen, R. A. Duursma, R. López, B. E. Medlyn, Triggers of tree mortality under drought. *Nature* **558**, 531–539 (2018).

9. T. J. Brodribb, J. Powers, H. Cochard, B. Choat, Hanging by a thread? Forests and drought. *Science* **368**, 261–266 (2020).

10. Y. J. Chen, B. Choat, F. Sterck, P. Maenpuen, M. Katabuchi, S. B. Zhang, K. W. Tomlinson, R. S. Oliveira, Y. J. Zhang, J. X. Shen, K. F. Cao, S. Jansen, Hydraulic prediction of drought-induced plant dieback and top-kill depends on leaf habit and growth form. *Ecol. Lett.* **24**, 2350–2363 (2021).

11. N. G. McDowell, G. Sapes, A. Pivovaroff, H. D. Adams, C. D. Allen, W. R. L. Anderegg, M. Arend, D. D. Breshears, T. Brodribb, B. Choat, H. Cochard, M. de Cáceres, M. G. de Kauwe, C. Grossiord, W. M. Hammond, H. Hartmann, G. Hoch, A. Kahmen, T. Klein, D. S. Mackay, M. Mantova, J. Martínez-Vilalta, B. E. Medlyn, M. Mencuccini, A. Nardini, R. S. Oliveira, A. Sala, D. T. Tissue, J. M. Torres-Ruiz, A. M. Trowbridge, A. T. Trugman, E. Wiley, C. Xu, Mechanisms of woody-plant mortality under rising drought, CO2 and vapour pressure deficit. *Nat. Rev. Earth Environ.* **3**, 294–308 (2022).

12. N. McDowell, W. T. Pockman, C. D. Allen, D. D. Breshears, N. Cobb, T. Kolb, J. Plaut, J. Sperry, A. West, D. G. Williams, E. A. Yepez, Mechanisms of plant survival and mortality during drought: Why do some plants survive while others succumb to drought? *New Phytol.* **178**, 719–739 (2008).

13. W. M. Hammond, K. Yu, L. A. Wilson, R. E. Will, W. R. L. Anderegg, H. D. Adams, Dead or dying? Quantifying the point of no return from hydraulic failure in drought-induced tree mortality. *New Phytol.* **223**, 1834–1843 (2019).

14. C. E. Doughty, D. B. Metcalfe, C. A. J. Girardin, F. F. Amézquita, D. G. Cabrera, W. H. Huasco, J. E. Silva-Espejo, A. Araujo-Murakami, M. C. da Costa, W. Rocha, T. R. Feldpausch, A. L. M. Mendoza, A. C. L. da Costa, P. Meir, O. L. Phillips, Y. Malhi, Drought impact on forest carbon dynamics and fluxes in Amazonia. *Nature* **519**, 78–82 (2015).

15. A. Sala, D. R. Woodruff, F. C. Meinzer, Carbon dynamics in trees: Feast or famine? *Tree Physiol.* **32**, 764–775 (2012).

16. B. T. Wolfe, Bark water vapour conductance is associated with drought performance in tropical trees. *Biol. Lett.* **16**, 20200263 (2020).

17. B. T. Wolfe, J. S. Sperry, T. A. Kursar, Does leaf shedding protect stems from cavitation during seasonal droughts? A test of the hydraulic fuse hypothesis. *New Phytol.* **212**, 1007–1018 (2016).

18. U. Hacke, J. Sperry. (Springer, 2001).

19. H. D. Adams, M. J. B. Zeppel, W. R. L. Anderegg, H. Hartmann, S. M. Landhäusser, D. T. Tissue, T. E. Huxman, P. J. Hudson, T. E. Franz, C. D. Allen, L. D. L. Anderegg, G. A. Barron-Gafford, D. J. Beerling, D. D. Breshears, T. J. Brodribb, H. Bugmann, R. C. Cobb, A. D. Collins, L. T. Dickman, H. Duan, B. E. Ewers, L. Galiano, D. A. Galvez, N. Garcia-Forner, M. L. Gaylord, M. J. Germino, A. Gessler, U. G. Hacke, R. Hakamada, A. Hector, M. W. Jenkins, J. M. Kane, T. E. Kolb, D. J. Law, J. D. Lewis, J. M. Limousin, D. M. Love, A. K. Macalady, J. Martínez-Vilalta, M. Mencuccini, P. J. Mitchell, J. D. Muss, M. J. O'Brien, A. P. O'Grady, R. E. Pangle, E. A. Pinkard, F. I. Piper, J. A. Plaut, W. T. Pockman, J. Quirk, K. Reinhardt, F.

Ripullone, M. G. Ryan, A. Sala, S. Sevanto, J. S. Sperry, R. Vargas, M. Vennetier, D. A. Way, C. Xu, E. A. Yepez, N. G. McDowell, A multi-species synthesis of physiological mechanisms in drought-induced tree mortality. *Nat. Ecol. Evol.* **1**, 1285–1291 (2017).

20. W. R. Anderegg, T. Klein, M. Bartlett, L. Sack, A. F. A. Pellegrini, B. Choat, S. Jansen, Meta-analysis reveals that hydraulic traits explain cross-species patterns of drought-induced tree mortality across the globe. *Proc. Natl. Acad. Sci.U.S.A.* **113**, 5024–5029 (2016).

21. J. S. Powers, G. Vargas, T. J. Brodribb, N. B. Schwartz, D. Pérez-Aviles, C. M. Smith-Martin, J. M. Becknell, F. Aureli, R. Blanco, E. Calderón-Morales, J. C. Calvo-Alvarado, A. J. Calvo-Obando, M. M. Chavarría, D. Carvajal-Vanegas, C. D. Jiménez-Rodríguez, E. Murillo Chacon, C. M. Schaffner, L. K. Werden, X. Xu, D. Medvigy, A catastrophic tropical drought kills hydraulically vulnerable tree species. *Glob. Chang. Biol.* **26**, 3122–3133 (2020).

22. M. D. Venturas, H. N. Todd, A. T. Trugman, W. R. Anderegg, Understanding and predicting forest mortality in the western United States using long-term forest inventory data and modeled hydraulic damage. *New Phytol.* **230**, 1896–1910 (2021).

23. R. Zweifel, F. Sterck, A conceptual tree model explaining legacy effects on stem growth. *Front. For. Glob. Change.* **1**, 00009 (2018).

24. N. K. Ruehr, R. Grote, S. Mayr, A. Arneth, Beyond the extreme: Recovery of carbon and water relations in woody plants following heat and drought stress. *Tree Physiol.* **39**, 1285–1299 (2019).

25. F. Magnani, M. Mencuccini, J. Grace, Age-related decline in stand productivity: The role of structural acclimation under hydraulic constraints. *Plant Cell Environ.* **23**, 251–263 (2000).

26. Y. Song, F. Sterck, U. Sass-Klaassen, C. Li, L. Poorter, Growth resilience of conifer species decreases with early, long-lasting and intense droughts but cannot be explained by hydraulic traits. *J. Ecol.* **110**, 2088–2104 (2022).

27. L. DeSoto, M. Cailleret, F. Sterck, S. Jansen, K. Kramer, E. M. R. Robert, T. Aakala, M. M. Amoroso, C. Bigler, J Julio Camarero, K. Čufar, G. Gea-Izquierdo, S. Gillner, L. J. Haavik, A.-M. Hereş, J. M. Kane, V. I. Kharuk, T. Kitzberger, T. Klein, T. Levanič, J. C. Linares, H. Mäkinen, W. Oberhuber, A. Papadopoulos, B. Rohner, G. Sangüesa-Barreda, D. B. Stojanovic, M. L. Suárez, R. Villalba, J. Martínez-Vilalta, Low growth resilience to drought is related to future mortality risk in trees. *Nat. Commun.* **11**, 545 (2020).

28. Y. Song, F. Sterck, X. Zhou, Q. Liu, B. Kruijt, L. Poorter, Drought resilience of conifer species is driven by leaf lifespan but not by hydraulic traits. *New Phytol.* **235**, 978–992 (2022).

29. L. Plavcová, G. Hoch, H. Morris, S. Ghiasi, S. Jansen, The amount of parenchyma and living fibers affects storage of nonstructural carbohydrates in young stems and roots of temperate trees. *Am. J. Bot.* **103**, 603–612 (2016).

30. V. B. Möhring, A. Bitter, G. Bub, M. Dieter, M. Dög, M. Hanewinkel, N. G. von Hatzfeldt, J. Köhler, G. Ontrup, R. Rosenberger, B. Seintsch, F. Thoma, Schadenssumme insgesamt 12,7 Mrd. Euro: Abschätzung der ökonomischen Schäden der Extremwetterereignisse der Jahre 2018 bis 2020 in der Forstwirtschaft. *Forstwirtschaft* **9**, 155–158 (2021).

31. W. Peters, A. Bastos, P. Ciais, A. Vermeulen, A historical, geographical and ecological perspective on the 2018 European summer drought. *Philos. Trans. R. Soc. B* **375**, 20190505 (2020).

32. Y. Song, L. Poorter, A. Horsting, S. Delzon, F. Sterck, Pit and tracheid anatomy explain hydraulic safety but not hydraulic efficiency of 28 conifer species. *J. Exp. Bot.* **73**, 1033–1048 (2022).

33. B. Choat, S. Jansen, T. J. Brodribb, H. Cochard, S. Delzon, R. Bhaskar, S. J. Bucci, T. S. Feild, S. M. Gleason, U. G. Hacke, A. L. Jacobsen, F. Lens, H. Maherali, J. Martínez-Vilalta, S. Mayr, M. Mencuccini, P. J. Mitchell, A. Nardini, J. Pittermann, R. B. Pratt, J. S. Sperry, M. Westoby, I. J. Wright, A. E. Zanne, Global convergence in the vulnerability of forests to drought. *Nature* **491**, 752–755 (2012).

34. J. Martínez-Vilalta, A. Sala, J. Piñol, The hydraulic architecture of Pinaceae–a review. *Plant Ecol.* **171**, 3–13 (2004).

35. D. M. Johnson, K. A. McCulloh, D. R. Woodruff, F. C. Meinzer, Hydraulic safety margins and embolism reversal in stems and leaves: Why are conifers and angiosperms so different? *Plant Sci.* **195**, 48–53 (2012).

36. C. Körner, No need for pipes when the well is dry—a comment on hydraulic failure in trees. *Tree Physiol.* **39**, 695–700 (2019).

37. M. Arend, R. M. Link, R. Patthey, G. Hoch, B. Schuldt, A. Kahmen, Rapid hydraulic collapse as cause of drought-induced mortality in conifers. *Proc. Natl. Acad. Sci.U.S.A.* **118**, e2025251118 (2021).

38. W. Wang, B. Vinocur, A. Altman, Plant responses to drought, salinity and extreme temperatures: Towards genetic engineering for stress tolerance. *Planta* **218**, 1–14 (2003).

39. R. Teskey, T. Wertin, I. Bauweraerts, M. Ameye, M. A. McGuire, K. Steppe, Responses of tree species to heat waves and extreme heat events. *Plant Cell Environ.* **38**, 1699–1712 (2015).

40. P. Krokene, Conifer Defense and Resistance to Bark Beetles, in *Bark Beetles* (Elsevier, 2015), pp. 177–207.

41. P. Reich, M. Walters, B. Kloeppel, D. Ellsworth, Different photosynthesis-nitrogen relations in deciduous hardwood and evergreen coniferous tree species. *Oecologia* **104**, 24–30 (1995).

42. C. Fortunel, C. Stahl, S. Coste, C. Ziegler, G. Derroire, S. Levionnois, I. Maréchaux, D. Bonal, B. Hérault, F. H. Wagner, L. Sack, J. Chave, P. Heuret, S. Jansen, G. John, C. Scoffoni, S. Trueba, M. K. Bartlett, Thresholds for persistent leaf photochemical damage predict plant drought resilience in a tropical rainforest. *New Phytol.* **239**, 576–591 (2023).

43. J. Margalef-Marrase, M. Á. Pérez-Navarro, F. Lloret, Relationship between heatwave-induced forest die-off and climatic suitability in multiple tree species. *Glob. Chang. Biol.* **26**, 3134–3146 (2020).

44. J. A. Sohn, S. Saha, J. Bauhus, Potential of forest thinning to mitigate drought stress: A meta-analysis. *For. Ecol. Manage.* **380**, 261–273 (2016).

45. T. J. Brodribb, D. J. Bowman, S. Nichols, S. Delzon, R. Burlett, Xylem function and growth rate interact to determine recovery rates after exposure to extreme water deficit. *New Phytol.* **188**, 533–542 (2010).

46. F. Sterck, L. Poorter, F. Schieving, Leaf traits determine the growth-survival trade-off across rain forest tree species. *Am. Nat.* **167**, 758–765 (2006).

47. I. Aleixo, D. Norris, L. Hemerik, A. Barbosa, E. Prata, F. Costa, L. Poorter, Amazonian rainforest tree mortality driven by climate and functional traits. *Nat. Clim. Chang.* **9**, 384–388 (2019).

48. L. Rowland, A. C. L. da Costa, D. R. Galbraith, R. S. Oliveira, O. J. Binks, A. A. R. Oliveira, A. M. Pullen, C. E. Doughty, D. B. Metcalfe, S. S. Vasconcelos, L. V. Ferreira, Y. Malhi, J.

Grace, M. Mencuccini, P. Meir, Death from drought in tropical forests is triggered by hydraulics not carbon starvation. *Nature*. **528**, 119–122 (2015).

49. R. S. Oliveira, C. B. Eller, F. V. Barros, M. Hirota, M. Brum, P. Bittencourt, Linking plant hydraulics and the fast–slow continuum to understand resilience to drought in tropical ecosystems. *New Phytol.* **230**, 904–923 (2021).

50. D. Ackerly, Functional strategies of chaparral shrubs in relation to seasonal water deficit and disturbance. *Ecological monographs* **74**, 25–44 (2004).

51. J. L. Quero, F. J. Sterck, J. Martínez-Vilalta, R. Villar, Water-use strategies of six co-existing Mediterranean woody species during a summer drought. *Oecologia* **166**, 45–57 (2011).

52. A. L. Pivovaroff, S. C. Pasquini, M. E. de Guzman, K. P. Alstad, J. S. Stemke, L. S. Santiago, Multiple strategies for drought survival among woody plant species. *Funct. Ecol.* **30**, 517–526 (2016).

53. A. T. Trugman, L. D. Anderegg, W. R. Anderegg, A. J. Das, N. L. Stephenson, Why is tree drought mortality so hard to predict? *Trends Ecol. Evol.* **36**, 520–532 (2021).

54. L. Rowland, J. Martínez-Vilalta, M. Mencuccini, Hard times for high expectations from hydraulics: Predicting drought-induced forest mortality at landscape scales remains a challenge. *New Phytol.* **230**, 1685–1687 (2021).

55. H. Cornelissen, U. Sass-Klaassen, L. Poorter, K. van Geffen, R. S. P. van Logtestijn, J. van Hal, L. Goudzwaard, F. J. Sterck, R. K. W. M. Klaassen, G. T. Freschet, A. van der Wal, H. Eshuis, J. Zuo, W. de Boer, T. Lamers, M. Weemstra, V. Cretin, R. Martin, J. den Ouden, M. P. Berg, R. Aerts, G. M. J. Mohren, M. M. Hefting, Controls on coarse wood decay in temperate tree species: Birth of the LOGLIFE experiment. *Ambio* **41**, 231–245 (2012).

56. TNO-NITG, www.cinoloket.nl [accessed January 2020].

57. J. J. H. Willinge Gratama-Oudemans, The arboretum of Schovenhorst, Putten, in the Netherlands. *Arboric. J.* **16**, 197–205 (1992).

58. Y. Song, U. Sass-Klaassen, F. Sterck, L. Goudzwaard, L. Akhmetzyanov, L. Poorter, Growth of 19 conifer species is highly sensitive to winter warming, spring frost and summer drought. *Ann. Bot.* **128**, 545–557 (2021).

59. V. Vitali, U. Büntgen, J. Bauhus, Silver fir and Douglas fir are more tolerant to extreme droughts than Norway spruce in south-western Germany. *Glob. Chang. Biol.* **23**, 5108–5119 (2017).

60. M. van der Maaten-Theunissen, E. van der Maaten, O. Bouriaud, pointRes: An R package to analyze pointer years and components of resilience. *Dendrochronologia* **35**, 34–38 (2015).

61. R. L. Peters, P. Groenendijk, M. Vlam, P. A. Zuidema, Detecting long-term growth trends using tree rings: A critical evaluation of methods. *Glob. Chang. Biol.* **21**, 2040–2054 (2015).

62. S. M. Vicente-Serrano, S. Beguería, J. I. López-Moreno, A multiscalar drought index sensitive to global warming: The standardized precipitation evapotranspiration index. *J. Climate* **23**, 1696–1718 (2010).

63. S. Begueria, S. Vicente-Serrano, Calculation of the Standardized Precipitation Evapotranspiration INdex (2013); SPEI R package version 1.

64. S. Delzon, C. Douthe, A. Sala, H. Cochard, Mechanism of water-stress induced cavitation in conifers: Bordered pit structure and function support the hypothesis of seal capillary-seeding. *Plant Cell Environ.* **33**, 2101–2111 (2010).

65. X. Wu, H. Liu, X. Li, P. Ciais, F. Babst, W. Guo, C. Zhang, V. Magliulo, M. Pavelka, S. Liu, Y. Huang, P. Wang, C. Shi, Y. Ma, Differentiating drought legacy effects on vegetation growth over the temperate Northern Hemisphere. *Glob. Chang. Biol.* **24**, 504–516 (2018).

66. R Core Team, *R: A Language and Environment for Statistical Computing* (R Foundation for Statistical Computing, 2021) version 4.1. 2; www.R-project.org/.

67. M. Weemstra, B. Eilmann, U. G. Sass-Klaassen, F. J. Sterck, Summer droughts limit tree growth across 10 temperate species on a productive forest site. *For. Ecol. Manage.* **306**, 142–149 (2013).

68. A. Farjon, D. Filer, *An Atlas of the World's Conifers: An Analysis of Their Distribution, Biogeography, Diversity and Conservation Status* (Brill, 2013).
